# Patterns of Care and Treatment Outcomes Among Men Diagnosed with Prostate Cancer from Culturally and Linguistically Diverse Backgrounds: A Scoping Review

**DOI:** 10.1007/s11912-025-01660-8

**Published:** 2025-03-29

**Authors:** Koku Sisay Tamirat, Michael James Leach, Nathan Papa, Jeremy Millar, Eli Ristevski

**Affiliations:** 1 School of Rural Health, Monash University, 15 Sargeant St, Warragul, VIC 3820 Australia; 2 School of Rural Health, Monash University, Bendigo, Australia; 3 School of Public Health and Preventive Medicine, Monash University, Melbourne, Australia; 4 School of Translational Medicine, Monash University, Melbourne, Australia; 5 Radiation Oncology, Alfred Health, Melbourne, Australia

**Keywords:** Prostate cancer, Patterns of care, Treatment outcomes, And culturally and linguistically diverse backgrounds

## Abstract

**Introduction:**

Men from culturally and linguistically diverse (CALD) backgrounds face challenges in accessing equitable and quality healthcare. However, little is known about the patterns of care among men diagnosed with prostate cancer (PCa) from CALD backgrounds. We aimed to map the available literature on patterns of care and treatment outcomes in men from CALD backgrounds who have PCa.

**Methods:**

We used the Johanna Briggs Institute scoping review methodology. We searched five bibliographic databases (Ovid MEDLINE, EMBASE, SCOPUS, CINAHL, and Ovid Emcare) and grey literature. We explored patterns of PCa care extending from screening and early detection to end-of-life care and treatment outcomes.

**Results:**

A total of 7,148 records were identified; 58 studies were included. Most studies were from the United States (US) (*n* = 41) and used ethnic origin (*n* = 14), nativity (*n* = 10), immigration history (*n* = 11), or country of birth (*n* = 13) as indicators of CALD. Most studies focused on screening and early detection for PCa (*n* = 37), specifically prostate-specific antigen (PSA) testing. Twelve papers were on PCa treatment (e.g., surgery, radiation therapy, and active surveillance), five on follow-up and supportive care, and four on treatment outcomes (i.e., change in measured PSA and PCa cancer-specific survival). There were disparities in the PCa care continuum and treatment outcomes between CALD and non-CALD patients. Factors influencing screening and early detection for PCa were systematically summarised and most addressed individual-level determinants.

**Conclusions:**

Key findings from our scoping review emphasised the existence of guideline-discordant care, disparities in PCa screening test use, and differences in PCa treatment received among men from CALD backgrounds. However, little is known about patterns of care in diagnostic modalities, treatment phases, and palliative and end-of-life care.

**Supplementary Information:**

The online version contains supplementary material available at 10.1007/s11912-025-01660-8.

## Introduction

In 2021, approximately 281 million migrants, representing 3.6% of the world’s population, lived outside their country of origin [1]. This migration is particularly notable in high Human Development Index (HDI) countries, where foreign-born individuals represent a substantial proportion of the total population: 29.3% in Australia, 21% in Canada, 14% in the United Kingdom (UK), and 14% in the United States (US) [2, 3]. Census data from 2021 and 2022 indicate that around 22% of the Australian and the US population spoke languages other than English at home [4, 5]. Immigrants bring a wide range of cultures, languages, religious beliefs and cultural practices that differ from those of a society's predominant cultural or linguistic groups, enriching its cultural fabric. Immigrant groups who share similar cultural and ethnic backgrounds, although not all are heterogeneous. Significant variations exist due to migration-related factors such as migration pathway, age at migration, length of time since migration, and duration of residence in the host country [6]. For this reason, the World Health Organization (WHO) has developed competency standards for healthcare workers for the delivery of quality care to migrants and refugees, which are aligned with an individual's cultural and linguistic contexts [7].

Various terms and categories are used to define migrants/immigrants and cultural and linguistically diverse (CALD) populations across different countries [8]. These include Black and Asia Minority Ethnic (BAME) in the UK [9], ethnocultural diversity and immigrants in Canada [10], immigrants/foreign-born and racial and ethnic minorities in the US [11] and Culturally and Linguistically Diverse (CALD) populations in Australia [12]. Common to these definitions is the focus on the country of birth and the main language spoken at home. These factors significantly influence exposure to risk factors, access to and utilisation of social and health services, as well as health outcomes [6, 13].

Disparities in cancer mortality and survival are evident among people from CALD backgrounds [14, 15]. Prostate cancer (PCa) is the most common cancer diagnosed in men. There were an estimated 1.4 million new PCa cases and 375,000 deaths worldwide in 2020, and these figures have been projected to approximately double by 2040 [16]. PCa and its management significantly affect physical, sexual, emotional and psychosocial well-being, impacting the quality of life [17, 18]. Growing evidence suggests that men with CALD backgrounds are presenting with advanced PCa and receiving suboptimal and guideline-discordant PCa care [19–22]. For instance, men with no English proficiency in the US had 44% lower odds of receiving PSA testing [20]. Research in the UK also demonstrated that black men with high-risk or locally advanced PCa had less access to radical local treatment than Whites [23]. Limited knowledge of PCa, fear of diagnosis, physical modesty, language barriers, socioeconomic disadvantage, and a preference for Eastern medicine were barriers to PCa healthcare services utilisations among CALD men [24–28].

To address these challenges, clinical pathways, best practice, and optimal care pathways have been developed in the US [29], UK [30] and Australia [31] for PCa care. These pathways recognise the continuum of care for PCa from screening to palliative and end-of-life care, aiming to enhance quality, efficiency, and patient-centeredness of healthcare delivery. By implementing clinical pathways, healthcare systems and services can better address the disparities in access and quality of care that affect CALD populations and ensure CALD populations receive equitable, effective, and culturally appropriate services, ultimately improving cancer outcomes [16, 31]. Evidence regarding the PCa care continuum for CALD populations is limited, making it challenging to understand their PCa care experiences and treatment outcomes. In light of this gap, we conducted a scoping review to answer the following research questions:

1. Which guideline-recommended screening and diagnostic methods are most commonly used among men with PCa from CALD backgrounds?
2. What are the barriers and facilitators to the utilisation of PCa screening and diagnostic services among men from CALD backgrounds?
3. What types of care and treatment (i.e., surgery, radiation therapy, chemotherapy, hormonal therapy, active surveillance/watchful waiting [AS/WW], and palliative care) have been provided to men with PCa from CALD backgrounds, and how frequently were each provided?
4. What are the treatment outcomes (i.e., PCa-specific mortality, PCa-specific survival, and biochemical recurrence) among men with PCa from CALD backgrounds?

A comprehensive understanding of patterns of PCa care and treatment outcomes among CALD men may improve the body of knowledge and guide future research endeavours in this area.

## Methods

This review is guided by the Joanna Briggs Institute (JBI) scoping review methodology [32] and reported according to the Preferred Reporting Items for Systematic Reviews and Meta-analysis Extension for Scoping Reviews (PRISMA-ScR) [33]. The protocol was registered with the Open Science Framework (link: osf.io/nxr63).

### Eligibility Criteria

The JBI Participant, Concept, and Context framework was used to define the eligibility criteria for our review [32]. The participants of interest were men diagnosed with PCa. Studies with non-human or biological specimens and simulation models were excluded. Patterns of PCa care and PCa treatment outcomes were the concepts of interest and were based on the National Comprehensive Cancer Network (NCCN) guidelines for patients with early-stage PCa [34] and the Australian Optimal Care Pathway for Men with Prostate Cancer [31]. Studies were included if they reported on any of the following in relation to PCa: 1) screening, early detection or diagnosis (i.e., PSA test, digital rectal examination (DRE), imaging, and biopsy), 2) treatment (i.e., surgery, radiation therapy, hormonal therapy, chemotherapy, AS/WW, and palliative and end-of-life care), or 3) treatment/care outcomes (limited to PCa-specific survival and mortality, and biochemical recurrence). Studies were excluded if they focused on new test validation, evaluated non-standard care (e.g., early phase trials and complementary or alternative medicine), or were epidemiological estimates of mortality or survival without PCa treatment information.

CALD background is an umbrella term and the context of interest in this review. As terms describing CALD are inconsistent we defined it in one or more of the following: 1) born in a country other than the country/ies of the study setting/s as indicated by self-reported country of birth or immigration records, 2) spoken or preferred language different from the dominant language(s) of the country/ies where the study took place, or 3) self-identified ethnicity/ethnic origin suggestive of a country of birth other than the country/ies where the study took place. Papers that restricted the definition of CALD to Indigenous peoples, racial and ethnic groups (non-self-identified), and higher generations of ancestry groups (e.g., African Americans) were excluded from the CALD definition used in this review, as suggested by past work [35–37].

### Types of Evidence Sources

All study designs were included. The grey literature from national health departments and cancer societies/organisations was also reviewed. Expert commentaries, perspective papers and editorials, conference abstracts, literature reviews, book chapters, and supplements without full text were excluded.

### Search Strategies

An initial search was built with the support of an experienced librarian and tested in Ovid MEDLINE using a set of five gold-standard articles [21, 25, 38–40]. The final search was conducted in Ovid MEDLINE, EMBASE (Ovid), SCOPUS, Cumulative Index to Nursing and Allied Health Literature (CINAHL), and Emcare (Ovid). Grey literature was sourced from: the Australian Institute of Health and Welfare, Cancer Council Australia, Victorian Prostate Cancer Outcomes Registry (PCOR-Vic), Cancer Research United Kingdom, Health Canada, Canadian Cancer Society, International Agency for Research on Cancer/World Health Organization, American Cancer Society, and European Prostate Cancer Coalition. The searches were limited to studies published in the English language between January 1, 2005, and June 24, 2024. This time period was used because of migration pattern changes in recent decades [1] and due to PCa Gleason grading system modifications in September 2005 [41]. Supplementary File 1 provides the Ovid MEDLINE search strategy.

### Study Selection

The search results were imported into the reference management software EndNote X9 [42], deduplicated, and then imported into the systematic review software Covidence® [43] for article screening and data extraction. One author (KS) undertook title and abstract screening, and two authors (KS and ER) independently completed a full-text review of the articles using the selection criteria. Discrepancies in exclusions were settled through discussion and consensus decision-making.

### Data Extraction/Charting

Data extraction and charting were conducted using a data extraction template in Microsoft Excel. For each study, the following information was extracted: study overview, study characteristics (country, study design and setting, year of data collection, sample size and sampling methods, and data collection procedures), population characteristics (CALD indicators and age), and specific information on patterns of care (across the PCa care continuum and treatment modalities) and PCa treatment outcomes (biochemical recurrence, and PCa-specific survival and/or mortality). Indicators of CALD backgrounds may include terminologies such as immigration history, ethnicity or ethnic origin, country of birth, nativity/foreign-born status, preferred language, and any combination of these [8].

### Summarising and Reporting the Results

We described the characteristics of the included studies and compiled a narrative summary of PCa patterns of care and treatment outcomes by CALD indicators. In addition, we identified barriers and facilitators influencing screening and early detection, PCa treatment, and treatment outcomes.

## Results

**Fig. 1:**
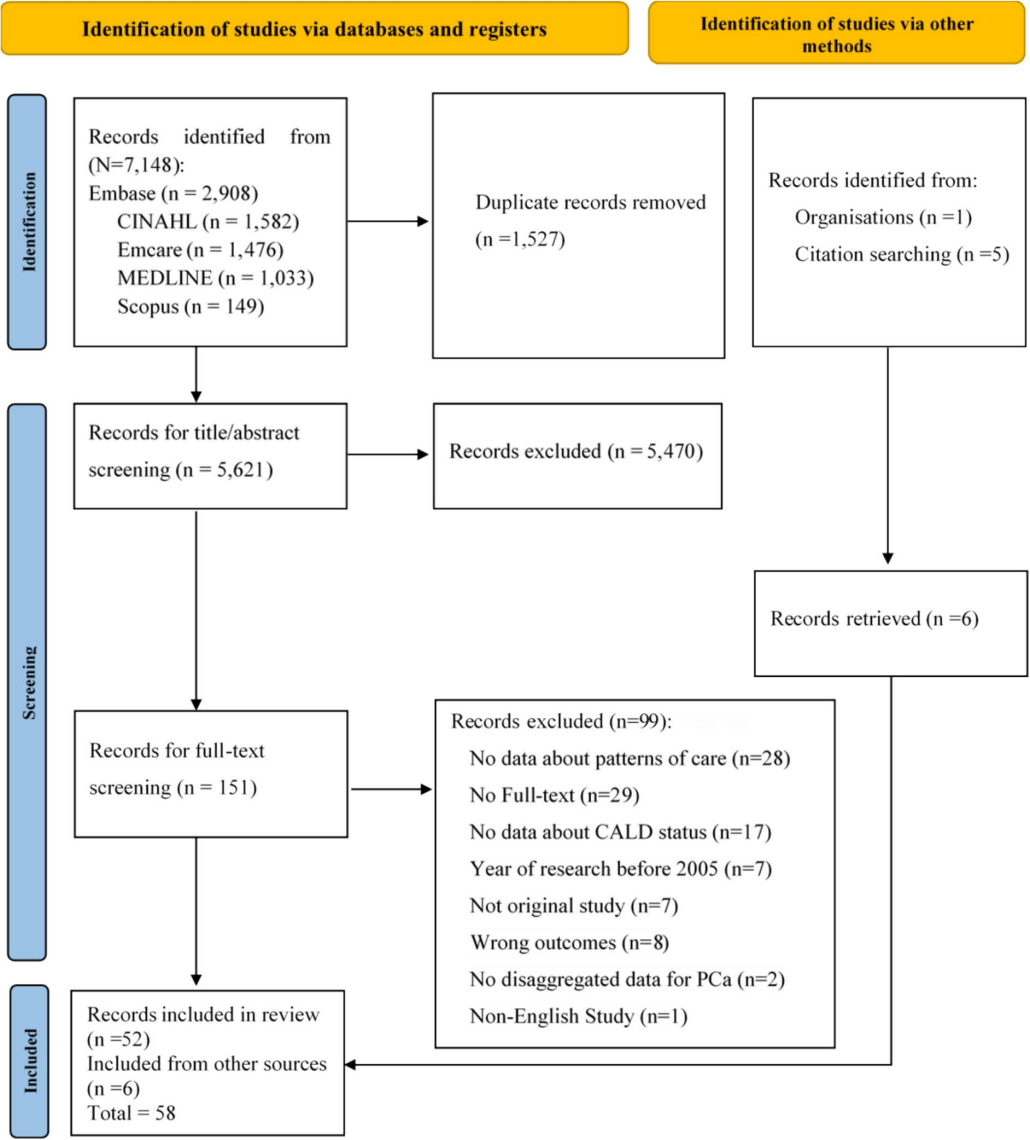
Preferred Reporting Items for Systematic Review and Meta-Analysis (PRISMA) flow diagram

A total of 7,148 records were identified. After removing duplicates and title/abstract/full-text screening, 58 papers were included in the review. Of these, six papers were found by manually reviewing the reference lists of included studies. Key reasons for exclusion were the absence of patterns of care data, lack of information regarding CALD indicators, and non-full-text articles (Fig. 1). Three Australian papers [21, 44, 45] used the same data source to answer different research questions, while four US papers [38, 46–48] used the same data.

### Study and Participant Characteristics

Most papers were from the US (*n* = 41) and used a cross-sectional study design (*n* = 24). Study sample sizes ranged from a minimum of seven [49] to a maximum of 2,061,047 [50] participants, with most studies (*n* = 31) recruiting < 1,000 participants. Data sources for quantitative studies were primary data collected by self- or interviewer-administered survey with/without additional medical record review (*n* = 33) and quantitative study data from secondary sources such as population-based cancer registries and hospital databases (*n* = 17). Data sources in the qualitative studies included interviews or focus group discussions (Table 1). Supplementary Table 1 provides further information on the included studies.

**Table 1:** Summary of study and participant characteristics (*n* = 58 papers)

Characteristics	Number of papers	Percentage	References
Country of study			
United States	41	70.7	[20, 24–28, 38–40, 46–48, 50–78]
United Kingdom	7	12.1	[49, 79–84]
Australia	6	10.3	[19, 21, 44, 45, 85, 86]
Norway	1	1.7	[87]
Sweden	1	1.7	[88]
Canada	1	1.7	[89]
Turkey	1	1.7	[90]
Study design			
Cross-sectional	24	41.4	[19–21, 24–27, 38, 39, 44–46, 54, 55, 59, 61, 62, 64–66, 68, 69, 72, 76]
Observational follow-up (cohort)	16	27.6	[40, 50, 51, 56–58, 60, 63, 67, 73, 75, 77, 78, 81, 87, 90]
Qualitative	8	13.8	[28, 49, 53, 71, 79, 83, 84, 89]
Mixed methods	5	8.6	[52, 70, 80, 82, 86]
Randomised control trial	3	5.2	[47, 48, 85]
Case–control	1	1.7	[88]
Ecological	1	1.7	[74]
Sample size of the studies			
< 1,000	31	53.4	[19, 24, 26, 28, 38, 39, 46–49, 51–54, 59–61, 65, 66, 68, 70, 71, 79, 80, 82–86, 89, 90]
1,000–4,999	5	8.6	[20, 39, 62, 69, 72]
≥ 5,000	22	37.9	[21, 25, 27, 40, 44, 45, 50, 55–58, 63, 64, 67, 73–78, 87, 88]
Data source and collection technique			
Quantitative study data from primary sources (self- or interviewer-administered survey, with/without medical record review)	33	56.9	[19–21, 24–27, 38, 39, 44–48, 51, 52, 54, 55, 59, 61, 62, 64–66, 68–70, 72, 76, 80, 82, 85, 86]
Quantitative study data from secondary sources (population-based registries and medical records)	17	29.3	[40, 50, 56–58, 60, 63, 67, 73–75, 77, 78, 81, 87, 88, 90]
Qualitative study data (interview or focus group discussion)	8	13.8	[28, 49, 53, 71, 79, 83, 84, 89]
Minimum age for inclusion			
< 50	22	38.0	[19, 24–26, 28, 38, 39, 44, 46–48, 50, 55, 61, 63–65, 67, 74, 76, 84, 86]
≥ 50	30	51.7	[20, 21, 27, 45, 51, 53, 54, 56–60, 62, 66, 68–71, 73, 75, 77, 78, 80–82, 85, 87–90]
Not reported/not clear	6	10.3	[40, 49, 52, 72, 79, 83]
Indicators of CALD backgrounds			
Ethnicity/ethnic origin	14	24.1	[19, 21, 27, 39, 46, 49–51, 53, 55, 58–60, 63, 64, 66, 68, 72, 73, 77, 79–82, 85, 87–89]
Foreign-born/nativity	10	17.2	[24, 25, 55, 61, 64, 67, 71, 73, 76, 78]
Country of birth	13	22.4	[21, 38, 39, 44, 52, 56–58, 62, 75, 82–84]
Immigration history	11	19.0	[26, 28, 40, 45, 47, 48, 54, 70, 87, 88, 90]
Preferred language other than English	6	10.3	[19, 20, 50, 51, 60, 85]
Combination of indicators	4	6.9	[65, 68, 69, 74, 86]
CALD population subgroups			
A mix of different CALD subpopulations	21	36.2	[19, 20, 25, 44, 45, 50–52, 55, 60, 61, 64, 66, 67, 74, 77, 80, 85–88]
Caribbean/African/Afro-Caribbean	19	32.8	[24, 38, 39, 46–49, 52, 54, 58, 65, 70–72, 79, 81–84]
Asian	11	19.0	[26, 28, 53, 59, 62, 63, 68, 69, 73, 78, 89]
Hispanic	6	10.3	[27, 40, 56, 57, 75, 76]
Syrian	1	1.7	[90]
Proportion of CALD men in each study’s sample			
< 0–24%	18	31.0	[19, 21, 39, 50, 51, 55–58, 60, 63, 64, 73, 81, 82, 85, 87, 88]
25%−49%	10	17.2	[20, 24, 44, 45, 61, 67, 71, 72, 77, 80]
50%−74%	7	12.1	[38, 40, 46, 52, 54, 70, 78]
75%−100%	20	34.5	[26–28, 47–49, 53, 59, 62, 65, 66, 68, 69, 75, 79, 83, 84, 86, 89, 90]
Not reported	3	5.2	[25, 74, 76]

*CALD* Culturally and Linguistically Diverse backgrounds

### Descriptors of Culturally and Linguistically Diversity

The most commonly used descriptors of CALD included ethnicity/ethnic origin (*n* = 13), nativity/foreign-born status (*n* = 10), country of birth (*n* = 13), and immigration history (*n* = 11). Caribbean and African/Afro-Caribbean (*n* = 19), Asian (*n* = 11), and Hispanic (*n* = 6) backgrounds were among the CALD subpopulations most represented in the studies. Six papers recruited participants who preferred to speak languages other than English (e.g., Spanish, French, Haitian-Creole, Cantonese, Toisanese, Mandarin, and other non-English Asian languages) or had limited English language proficiency [19, 20, 50, 51, 60, 85]. Four studies excluded men who had limited command of English [24, 49, 52, 79]; however, several other studies indicated that ethnically matched data collectors or research assistants, community leaders, and onsite translators were used as mitigating strategies to recruit and collect data from CALD men [26, 39, 53, 59, 62, 65, 68–71, 86].

### Overview of Patterns of Care and Treatment Outcomes

Most of the papers (*n* = 37) focused on screening and early detection for PCa, with a smaller number on treatment (*n* = 12) and follow-up and supportive care (*n* = 5). There were no papers on the utilisation of PCa diagnostic modalities or palliative and end-of-life care. There were four papers on treatment outcomes. (Table 2).

**Table 2:** Frequencies of papers on patterns of prostate cancer care and treatment outcomes by countries where studies were conducted (*n* = 58)

Country	Prostate cancer care continuum					OUTCOMES
	Screening and early detection (*n* = 37)	Diagnostic modalities (*n* = 0)	Treatment (*n* = 12)	Follow-up and supportive care (*n* = 5)	Palliative and end-of-life care (*n* = 0)	Treatment outcomes (*n* = 4)
United States	••••••••••••••••••••••••••• [20, 24–28, 38, 39, 46–48, 50, 52–55, 59, 62, 64–70, 72, 76]	--	••••••••• [51, 57, 58, 61, 63, 73–75, 78]	• [71]	--	•••• [40, 56, 60, 77]
Australia	•••••• [19, 21, 44, 45, 85, 86]	--	--	--	--	--
United Kingdom	••• [49, 80, 83]	--	• [81]	••• [79, 82, 84]	--	--
Canada	--	--		• [89]	--	--
Norway	--	--	• [87]	--	--	--
Sweden	• [88]	--	--	--	--	--
Turkey	--	--	• [90]	--	--	–

Dash (--) in cells denote no study is available

### Screening and Early Detection

Of the 37 papers on PCa screening and early detection, 25 focused on PSA testing. Screening and early detection for PCa were predominantly ascertained through self-reports (*n* = 25). Twenty-three papers reported PSA testing or DRE in the last 10 years of data collection. In 18 papers, men aged under 50 years were recruited for PCa screening and early detection; most of these papers (*n* = 15) were from the US (Table 3).

**Table 3:** Frequencies of papers on prostate cancer screening and early detection by culturally and linguistically diverse backgrounds indicators (*n* = 37)

Characteristics	Culturally and linguistically diverse backgrounds indicators					
	Immigration history (*n* = 8)	Ethnicity / ethnic origin (*n* = 9)	Country of birth (*n* = 7)	Nativity / Foreign-born (*n* = 6)	Preferred Language other than English (*n* = 4)	Combination of descriptors (*n* = 3)
Screening and early detection methods						
PSA testing (*n* = 25)	••••• [26, 45, 47, 54, 88]	••••• [46, 53, 59, 68, 27]	••• [21, 44, 62]	•••••• [24, 25, 55, 64, 67, 76]	••• [19, 20, 50]	••• [65, 69, 86]
DRE (*n* = 3)	• [28]	• [80]	• [38]	--	--	--
Both PSA and DRE (*n* = 5)	•• [48, 70]	• [66]	• [39]	--	• [85]	--
Not reported (*n* = 4)	-	•• [49, 72]	•• [52, 83]	--	--	--
Screening and early detection ascertained by						
Self-report (*n* = 25)	•••• [26, 45, 54, 70]	•••••• [46, 53, 59, 66, 68, 27]	•••• [21, 38, 39, 62]	•••••• [24, 25, 55, 64, 67, 76]	•• [20, 85]	••• [65, 69, 86]
Secondary data (*n* = 6)	••• [47, 48, 88]		• [44]	--	•• [19, 50]	--
Not reported (*n* = 6)	• [28]	••• [49, 72, 80]	•• [52, 83]	--	--	--
Screening and early detection status						
Recently screened ^a^ (*n* = 23)	•••••• [26, 45, 47, 48, 54, 70]	•••• [46, 59, 66, 27]	•••• [21, 38, 39, 44]	••• [24, 67, 76]	•••• [19, 20, 50, 85]	•• [69, 86]
Lifetime screening^b^ (*n* = 8)	• [88]	•• [53, 68]	• [62]	••• [25, 55, 64]	--	• [65]
Not reported (*n* = 6)	• [28]	••• [49, 72, 80]	•• [52, 83]	--	--	--
Screening and early detection adherence ‡ reported (*n* = 7)	• [54]	••• [46, 59, 27]	• [38]	• [76]	--	• [69]
Lowest bound age of inclusion for screening						
< 50 years (*n* = 18)	•••• [26, 28, 47, 48]	• [46]	••• [38, 39, 44]	•••••• [24, 25, 55, 64, 67, 76]	•• [19, 50]	•• [65, 86]
> 50 years (*n* = 15)	•••• [45, 54, 70, 88]	••••• [53, 59, 66, 68, 27]	•• [21, 62]	--	•• [20, 85]	• [69]
Not applicable (*n* = 4)	--	•• [49, 72]	•• [52, 83]	--	--	--
Studies with statistically significant differences in PCa screening service uptake (*N* = 18)	•••• [45, 54, 70, 88]	••• [46, 66, 80]	••• [21, 38, 44]	•••• [25, 55, 64, 67]	•••• [19, 20, 50, 85]	--

^a^Recently screened: PCa screening in the past and within ten years of the data collection

^b^Lifetime screened: papers which reported ever or never prostate cancer screening in the past

Not applicable: No screening data with age or unclear

DRE: Digital Rectal Examination,

CALD: Culturally and Linguistically Diverse

PSA: Prostate-Specific Antigen.

Screening adherence‡ refers to papers reporting regular PCa screening and early detection as per the guidelines and may not add up to the column total.

• papers reported differences or disparities in screening and early detection. Dash (–) in cells denotes no study is available

Twenty-one papers conducted comparative or stratified analyses of PCa screening and early detection service uptake between men from CALD and non-CALD backgrounds [19–21, 24, 25, 38, 39, 44–46, 50, 54, 55, 64, 66, 67, 70, 76, 80, 85, 88]. Of these, 15 papers reported that one or more CALD background subpopulations received statistically significantly less frequent screening for PCa compared to their non-CALD counterparts [19–21, 25, 38, 44, 46, 50, 54, 55, 64, 66, 80, 85, 88]. Three papers reported that CALD men underwent screening for PCa more frequently than non-CALD men [45, 67, 70]. Three papers reported no statistically significant differences in screening rates between CALD and non-CALD men [24, 39, 76] (Table 3 and Supplementary Table 2, which provides key findings along with a summary of statistics regarding screening and early detection).

Eight papers identified a statistically significant association between the ethnic characteristics of CALD men and PCa testing [25, 38, 46, 54, 55, 66, 70, 80] (Table 4 and supplementary Table 2). Of these, three US papers reported that men from Caribbean, Jamaican, or Trinidadian and Tobagonian backgrounds were more likely to initiate PSA testing or DRE compared to White men but were less likely to undergo or maintain annual screenings [38, 46, 70]. Seven papers set in the US (*n* = 6) and the UK (*n* = 1) found that men from Asian, Haitian, African, Trinidadian and Tobagonian, Caribbean, or Hispanic ethnic groups were less likely to undergo annual PSA testing or maintenance of annual DREs, compared to men from non-CALD backgrounds ( such as Non-Hispanic White and European-American men) [25, 38, 46, 54, 55, 66, 80]. For instance, a cross-sectional study in the US found that Haitian, Eastern European, and Puerto Rican men had statistically significantly fewer DREs in the past 10 years compared to European-American men. The average number of DREs was 1.6, 2.7, and 2.3 for these groups, respectively, compared to 3.1 for European-American men (*p* < 0.05) [66]. Another cross-sectional study from the US also reported that foreign-born Asians (23.9%) and Black/African Americans (45.83%) were less likely to get PSA tested than White Americans (56.3%), despite their increased risk of PCa due to a family history of cancer [25].

**Table 4:** Summary of factors associated with prostate cancer screening among men from culturally and linguistically diverse backgrounds (*n* = 25 quantitative papers)

Factors	Positively associated	Negatively associated
CALD-DEFINING (*N* = 17)		
Ethnicity/ethnic origin (*n* = 8)	▪ Caribbean immigrants [70] ▪ Jamaican, and Trinidadian and Tobagonian [38, 46]	▪ Foreign-born European non-Hispanic Whites [55] ▪ Jamaican or Trinidadian and Tobagonian background [38, 46, 54] ▪ Asian [25, 80], ▪ Foreign-born African American [25] ▪ Haitian and Puerto Rican [66]
Country/region of birth/ nativity/foreign-born (*n* = 8)	**--**	▪ Born in Europe [55] ▪ African immigrants [70, 88] ▪ Foreign-born [64] ▪ Born in China [62] ▪ North America, New Zealand, the UK or Ireland [44, 45] ▪ East Asia [21] ▪ Asia [88] ▪ The Middle East and North Africa [21, 88] ▪ Nordic countries other than Sweden (e.g., Denmark, Iceland, Finland, and Norway) [88] ▪ Central and South America [88] ▪ Eastern, Western, and Southern Europe [88]
Preferred language (*n* = 4)	**--**	▪ Preferred language other than English [19, 20, 62, 85]
SOCIODEMOGRAPHIC FACTORS (*N* = 10)		
Sociodemographic variables	▪ Increasing age [27] ▪ Age > 72 years [62] ▪ High school education [47] ▪ Associate degree education [25] ▪ Bachelor's degree education [25] ▪ Income USD 100,000 or over [25] ▪ Higher income in USD [27] ▪ Health insurance coverage [26, 27, 59, 68] ▪ Rural residence [45] ▪ Length of stay in years in the hosting countries [21, 62] ▪ Higher acculturation score [27]	▪ Younger age (40–49 years) [25] ▪ Employed [25]
KNOWLEDGE AND ATTITUDE (*N* = 3)		
Knowledge and attitude factors	▪ Low PCa knowledge associated with increased undisclosed screening [47] ▪ Perceived susceptibility to PCa [65]	▪ Physical modesty [26] ▪ Preference to use Eastern medicine [26] ▪ Crisis-oriented health-seeking behaviour [26]
HEALTHCARE UTILISATIONS AND CLINICAL FACTORS (*n* = 5)		
Health care services	▪ No plan to get tested for PCa associated with increased undisclosed screening [47] ▪ Self-reported health status [62] ▪ Seen a doctor in the last 12 months [25] ▪ Had regular sources of care [25] ▪ Had a personal physician [38] ▪ No physician recommendation associated with increased undisclosed screening [47] ▪ Availability of primary healthcare providers [68]	**--**
COMMUNITY LEVEL (*N* = 1)		
Social cohesion (*n* = 1)	▪ Medium neighbourhood social cohesion [62]	**--**
POLICY LEVEL (*N* = 1)		
Policy on health insurance (*n* = 1)	▪ The 2014 Affordable Care Act reform to immigrants and foreign-born individuals [67]	**--**

*PCa* Prostate cancer, *USD* United States Dollar, *UK* United Kingdom

Eight papers reported that men’s country/region of birth or nativity/foreign-born status were negatively associated with PSA testing compared to native-born or long-term resident men (Table 4 and Supplementary Table 2). These papers were set in Australia [21, 44, 45], the US [55, 62, 64, 70], and Sweden [88]. Notably, foreign-born men were less likely to undergo PSA testing than native-born men, with considerable variations by regions of birth; men from East Asia, the Middle East, Africa, the Caribbean, and Continental Europe demonstrated lower uptake compared to native-born in Sweden, the US, or Australia [44, 45, 62, 88].

Four papers, two from Australia [19, 85] and two from the US [20, 62], reported that men who spoke a language other than English or had limited English proficiency were associated with lower uptake of PCa screening (Table 4 and supplementary Table 2). The US study, which analysed 2,889 study participants (aged ≥ 55 years), found that men who had no English language proficiency (did not speak English at all) had 44% lower odds of undergoing a PSA test (aOR = 0.56, 0.35–0.91; *p* = 0.019), suggesting the existence of inequitable access to PSA testing [20].

### Screening and Early Detection Adherence

Seven papers, all from the US, provided data on adherence to PCa screening (i.e., repeat PSA testing/DRE every 1–2 years) (Table 3). Five papers indicated men from CALD backgrounds in the US exhibited less frequent compliance to guideline-recommended PCa screening [38, 46, 54, 59, 69]. For instance, a US study conducted among South Asians (Indian, Pakistani, Bangladeshi, and Sri Lankan) found that only one in ten South Asian males received guideline-adherent PCa screening [59]. Additional papers from the US also indicated that while CALD men had a greater tendency to initiate PSA testing, they were less likely to maintain annual examinations or tests [38, 46, 54] (supplementary Table 2).

### Barriers and Facilitators to Prostate Cancer Screening and Early Detection

Tables 4 and 5 present sociodemographic, knowledge and attitude, healthcare service, community, and policy factors that influence PCa screening and early detection among CALD men.

**Table 5:** Summary of barriers and facilitators to early detection and screening of prostate cancer among men from culturally and linguistically diverse backgrounds (*n* = 8 papers qualitative and mixed methods)

Facilitators	Barriers
Individual (*N* = 7)	
Sociodemographic ▪ Age [49] Responsibility to family and others ▪ Sense of responsibility to family and others [52] Personal or family history of cancer ▪ Personal experience or family history of cancer [53, 70] ▪ Influenced by family and friends [49] Symptoms ▪ Symptom recognition [80] ▪ Urinary symptoms [53] ▪ Disclosure of lower urinary tract symptoms [80] ▪ Opportunistic screening [49]	Sociodemographic ▪ Socioeconomic position [49] ▪ Financial issue [53] ▪ Time constraint [53] ▪ Lack of health insurance [52] Knowledge and attitude ▪ Fear of diagnosis [28, 53, 70] ▪ Lack of health education about PCa [49] ▪ Lack of knowledge about PCa [28, 52, 53] ▪ Misconceptions about PCa and incorrect correlation of PCa with the end of sexual life [52, 70] ▪ Fear of homophobia [70, 83] ▪ Intrusiveness and ambiguity on the necessity of the test [70] Screening habit ▪ No habit of regular health screening [53]
Healthcare utilisations and clinical factors (*N* = 6)	
Physician recommendations ▪ Recommendations and suggestions from providers [49, 53]	Gender of healthcare providers ▪ Gender of healthcare provider [86] Patient-physician communication ▪ Misinformation (inconsistent information from healthcare providers) [52] ▪ Poor GP-patient communication [80] ▪ Feeling of embarrassment [28, 53] Healthcare accessibility and trust ▪ Trust with the general practitioner GP [80] ▪ Healthcare affordability [52]
Community (*N* = 1)	
--	Cultural taboo ▪ Reservation to rectal examinations [28]

*GP* General Practitioner, *PCa* Prostate cancer

#### Sociodemographic

Age was found to influence PCa screening utilisation [25, 27, 49, 62]. Younger age (40–49 years) was associated with lower odds of PCa screening [25], while older age (median age ≥ 72 years) was associated with increased odds of PSA testing [27, 62]. One qualitative paper also indicated older age as a facilitator of screening for PCa [49].

A positive association was found between men’s level of education and PSA testing [25, 47]. CALD men with bachelor's/associate degrees had higher odds of getting PSA-tested than CALD men educated to high school level or less [25]. Conversely, male immigrants educated to high school or less were more likely to undergo undisclosed PSA testing (i.e., getting PSA-tested without informed consent and shared decision-making) compared to men who attended college or post-secondary [47].

Income or socioeconomic profile has also influenced PSA testing [25, 27, 49]. Men with a higher income or annual income above 100,000 US Dollars (USD) were more likely to undergo PSA testing than men with an annual income below 35,000 USD [25, 27]. Another qualitative paper identified men’s low socioeconomic position as a barrier to PCa screening, including PSA testing [49].

A positive correlation was also found between the possession of health insurance for men from CALD backgrounds and the receipt of PSA testing [26, 27, 59, 68]. Lack of health insurance coverage or financial difficulties were recognised as potential barriers to undergo PSA screening test [52, 53].

Finally, a higher acculturation score (i.e., measured in terms of linguistic and ethnic social relations) and duration of stay in countries of destination (e.g., the US and Australia) were associated with higher odds of receipt PSA test [21, 27, 62].

#### Knowledge and Attitudes

Eight papers examined knowledge- and attitude-related factors: two cross-sectional studies conducted in the US reported that the level of PCa knowledge and perceived susceptibility to PCa were associated with increased uptake of PSA testing in men from CALD backgrounds [47, 65]. One US cross-sectional study, which included 134 Korean immigrants, also identified that preferences for and use of Eastern medicine, physical modesty, and crisis-oriented health-seeking behaviour were associated with less frequent PSA testing [26]. Additionally, six qualitative papers identified that barriers to uptake of PCa screening included fear of diagnosis [28, 53, 70], lack of knowledge/understanding of PCa [28, 49, 52, 53], misconceptions about PCa and incorrect correlation of PCa with the end of sexual life [52, 70], shame and fear of homophobia [70, 83], ambiguity on the necessity of the PSA test [70], and lack of time to undertake PSA testing [53].

#### Health Care Service

Healthcare service utilisation and clinical factors also shaped screening and early detection for PCa in men from CALD backgrounds [28, 47, 49, 52, 53, 80, 86]. Three quantitative papers from the US and one from the UK have shown that physician recommendations for PSA testing increased/facilitated receipt of PSA test [47, 49, 53]. Four qualitative research papers highlighted that embarrassment [28, 53], poor patient-physician communication, and inconsistent health information provided by healthcare workers hindered the uptake of PCa screening in men from CALD backgrounds [52, 80]. In a mixed Australian study that involved 256 CALD men, the analysis indicated that gender of the healthcare provider was found to influence the uptake of PCa screening; 34% of CALD men preferred male health professionals, while 12% preferred female health professionals [86]. At the organizational level [52, 68, 80], access to primary healthcare providers has been reported to increase PSA testing [68]. Trust issues with general practitioners and healthcare affordability were also identified as barriers to PCa screening for men from CALD backgrounds [52, 80].

#### Community-Level Factors

Community-level factors also shaped the uptake of PSA testing [28, 62]. Chinese Americans living in a neighbourhood of medium-level cohesion were more likely to receive PSA testing than those who had no neighbourhood cohesion [62]. A qualitative study among male Indo-Guyanese immigrants in the US showed that cultural taboos (refusal) towards DRE hindered the timely detection and identification of PCa [28].

#### Policy Level

A US study reported the effects of the 2014 policy reforms of the Affordable Care Act on the uptake of preventive health services. Compared to continuously insured US-born men, newly insured male immigrants in the US were more likely to receive PSA tests [67].

### Prostate Cancer Treatment Modalities

Twelve papers reported data on PCa treatment; the majority were from the US [51, 57, 58, 61, 63, 73–75, 78], and the remaining three papers were from Norway [87], Turkey [90], and the UK [81]. Eight papers focused on the patient population diagnosed with localised and locally advanced PCa cases [51, 61, 63, 73–75, 81, 87], while the remaining four papers considered all PCa cases [57, 58, 78, 90]. In eight papers, there was evidence of variation in PCa treatment received between men from CALD and non-CALD backgrounds [57, 58, 61, 63, 74, 75, 78, 81] (Table 6).

**Table 6:** Evidence map illustrating patterns of prostate cancer treatment (*n* = 12)

	Culturally and linguistically diverse backgrounds indicators					
	Immigration history (*n* = 2)	Ethnicity / ethnic origin (*n* = 2)	Country of birth (*n* = 3)	Nativity / Foreign-born (*n* = 3)	Preferred language other than English (*n* = 1)	Combination of descriptors (*n* = 1)
Included patient groups						
Localised or locally advanced (*n* = 8)	• [87]	•• [63, 81]	• [75]	•• [61, 73]	• [51]	• [74]
Metastatic PCa (*n* = 0)	--	--	--	--	--	--
All PCa cases (*n* = 4)	• [90]	--	• • [57, 58]	• [78]	--	--
Treatment type						
Surgery only (*n* = 2)	--	--	• [58]	•• [73, 78]	--	--
Radiation therapy with or without ADT only (*n* = 1)	• [90]	--	--	--	--	--
Hormonal/ADT only (*n* = 0)	--	--	--	--	--	--
More than one aggressive local therapies (i.e., surgery, radiation, ADT, or any combinations) (*n* = 4)	• [87]	--	• [57]	• [61]	--	• [74]
Treatment received/ given (unspecified) (*n* = 2)	--	• [63]	• [75]	--	--	--
AS follow-up adherence (*n* = 2)	--	• [81]	--	--	• [51]	--
Studies with statistically significant treatment received/variations (*n* = 8)	--	•• [63, 81]	••• [57, 58, 75]	•• [61, 78]	--	• [74]

• papers reported data on PCa treatment, dash (--) denotes no study is available

*ADT* Androgen deprivation therapy, *AS* Active surveillance, *PCa* Prostate Cancer

Three papers examined surgical (prostatectomy) treatment of PCa [58, 73, 78]; two of these papers reported that CALD patients from foreign-born Asian, Jamaican, and West African backgrounds were more likely to receive surgery compared to their non-CALD counterparts [58, 78]. Additionally, men born in South Asia who underwent radical prostatectomy had poorer pathological profiles than the general population in the US [73].

Four papers have examined multiple definitive treatments or local aggressive therapies, including surgery, radiation therapy, or any combination of these treatment modalities [57, 61, 74, 87]. Findings showed a substantial difference in PCa treatment; Hispanics underwent more frequent External Beam Radiation Therapy (EBRT) than Non-Hispanic Whites (NHW); 46% of Cubans and 39% of Puerto Ricans versus 26% of NHW received EBRT [57]. NHW patients also received robot-assisted prostatectomy (RAP) more often than Hispanics: 37% in NHW versus 23% in Cubans and 26% in Puerto Ricans [57]. A Norwegian retrospective study, however, reported that while RAP was commonly performed for managing PCa for men under 75 years (59.8%), there was no statistically significant difference in utilisation rate between immigrants and native-born Norwegians [87]. Conversely, a retrospective analysis of 253 US men found that foreign-born blacks with intermediate-risk disease were more likely to receive surgery (55% versus 18%) and less likely to receive radiation therapy (35% versus 50%) compared to US-born men in the same risk group [61]. Another population-level study in the US highlighted that men living in counties with a greater number of foreign-born residents had been associated with increased odds of receiving aggressive local therapies (i.e., surgery or radiation therapy) [74].

Only one study assessed radiation therapy receipt and compliance among 137 Syrian refugees in Turkey, 65% of whom had locally advanced or metastatic PCa [90]. The research showed that conventional fractionated radiation therapy with infrequent ADT was used as the definitive treatment of PCa. Only 20% of patients received ADT alongside radiation therapy. Additionally, the study reported a higher non-compliance (defined as missing two or more fractions) rate (42%) to prescribed radiation doses among refugees [90].

Two papers reported active surveillance (AS) and related issues. Research from a high-volume centre in the UK revealed that African/Afro-Caribbean men were significantly more likely to have non-attendance to AS follow-up (24%) compared with 10% in Caucasians/others [81]. The same study also showed that, over time, the proportion of men remaining on AS decreased significantly. Between the 24th and 60th months, the percentage of men remaining on AS dropped from 89 to 67% among African/Afro-Caribbean men and from 88 to 63% in Caucasians/others. A different US study involving 713 participants, 11% of whom spoke languages other than English, found no significant difference in choosing AS over immediate active treatment based on the preferred spoken language [51].

Two other US papers reported on unspecified PCa treatment (active treatment or AS received versus no treatment/AS received) among Asian Americans and Hispanics [63, 75] (Table 6). Both groups were more likely to present with high-risk PCa upon diagnosis; however, Hispanics were less likely to receive PCa treatment [75]. In contrast, Japanese Americans had greater odds of receiving PCa treatment at diagnosis compared to White and Chinese men; disparities remained evident in a subgroup disaggregated analysis of Asian patients [63] (Table 6 and supplementary Table 3).

### Factors Influencing the Treatment of Prostate Cancer

While six papers examined the role of CALD indicators in PCa treatment utilisation [57, 61, 63, 74, 75, 81], none reported the interplay of socioeconomic, behavioural, disease-related, or health system factors influencing PCa treatment among CALD patients. One UK qualitative study found that PCa survivors’ perspectives on whether to opt for a particular PCa treatment were shaped by the interaction of clinical, behavioural, masculinity, and cultural factors [79]. Two other studies from the US and Turkish demonstrated PCa treatment variations and compliance based on place of residence [74, 90]. The US study revealed that men living in a borough with more proportions of foreign-born residents were more likely to receive aggressive local therapies [74]. The Turkish study also indicated that patients who were living in refugee camps had a higher rate of treatment non-compliance compared to those living in houses (64% vs. 30%) [90].

### Follow-up and Supportive Care

Five papers examined life experiences during and after PCa diagnosis and treatment [71, 79, 82, 84, 89]. Three of these papers were from the UK and used qualitative or mixed methods approaches [79, 82, 84]. Two papers indicated that PCa survivors sought psychosocial support from local community networks, such as churches and country-of-birth associations [82, 84]. Survivors also received post-treatment informal care from family members and local community organisations, including PCa support groups [82, 84, 89].

Two papers reported that PCa survivors navigated information regarding their disease condition and its effect from peers with similar diagnoses, medical practitioners who were family or friends, and online resources [79, 89]. A study involving Chinese Canadians indicated that survivors had limited access to health information from their clinicians and often verified the information they received with external sources [89].

Regarding the healthcare experiences, two papers highlighted that PCa survivors perceived healthcare systems as unresponsive and lacking cultural sensitivity, leading to fragmented care and poor coordination between services and agencies [84, 89]. Moreover, survivors reported receiving inadequate information regarding self-management of treatment-related side effects (e.g., incontinence) [84].

Two qualitative studies from the US [71] and Canada [89] also explored survivorship experiences, highlighting that men require psycho-oncology and emotional support at the point of care and during subsequent follow-up. The US study highlighted that men encountered fear, denial, disbelief, overwhelmingness, and “why me” syndrome at the point of PCa diagnosis, underscoring the necessity for psycho-oncological and emotional support related to their PCa diagnosis [71]. The Canadian study also indicated that PCa survivors had unmet needs for social support, emotional support, as well as coping strategies related to the disease’s impact. Moreover, follow-up care inadequately addressed the mental and emotional aspects of the disease and its treatment [89]. The same study also found that telehealth follow-up could disrupt care transition and affect the quality of patient-clinician relationships. In addition, PCa survivors suggested that PCa follow-up care should include traditional Chinese medicine [89].

### Treatment Outcomes

Only four papers from the US reported PCa treatment outcomes [40, 56, 60, 77]. These papers focused on biochemical recurrence [60] and PCa-specific survival and mortality after PCa treatment [40, 56, 77]. A retrospective cohort study found that after EBRT, Haitian-Creole-speaking PCa patients had a statistically significantly greater change in serum PSA levels from pre-to post-treatment (indicating likely biochemical recurrence) compared to English-speaking patients (PSA change; 5.8 vs 8.7 ng/ml, *p* = 0.039) [60]. Another cohort study found that foreign-born Hispanic men with PCa living in high-ethnic (HR = 1.04, 95%CI: 1.04–1.06) or low-ethnic enclaves (HR = 1.05, 95%CI:104–1.06) had better PCa-specific survival compared to US-born men [40]. Conversely, Puerto Rican men treated with prostatectomy had worse PCa-specific mortality than NHW who underwent the same treatment (HR = 2.55, *p* = 0.004) [77]. Another US study found statistically significant differences in PCa-specific survival among Hispanic men based on their country of birth: compared to men of Mexican origin, those of Dominican (HR = 0.69, 95% CI: 0.53–0.97; *p* = 0.034), South and Central American (HR = 0.78, 95% CI: 0.71–0.87; *p* < 0.001), and non-specified Hispanic origins (HR = 0.75, 95% CI: 0.71–0.80; *p* < 0.001) had better survival [56]. However, none of these studies examined the roles of socioeconomic, demographic, provider, and health system factors on PCa survival outcomes among men from CALD backgrounds.

## Discussion

Our scoping review identified 58 papers examining patterns of PCa care and treatment outcomes involving men from CALD backgrounds. The vast majority of these studies were from the US and provided data on screening and early detection for PCa, particularly PSA testing. Overall, the review highlighted that men from CALD groups had poor access to screening and early detection services for PCa. While these studies provide valuable insights into the continuum of PCa care and treatment outcomes, substantial gaps remain in understanding PCa diagnostic methods, treatment phases, and palliative and end-of-life care for men from CALD backgrounds.

### Definitions to Measure Cultural and Linguistic Diversity

While there is no universally accepted definition of CALD status, researchers commonly use various sociodemographic characteristics to designate CALD status. These included country of birth, immigration status, nativity status, ethnic origin, preferred language spoken, or any combination of these. Considering these indicators in study design, analysis, and reporting can help to identify groups experiencing inequities in the PCa care continuum and outcomes [91]. However, some categorisations, such as nativity status (foreign-born versus native-born) without further disaggregation, may obscure or average out differences within CALD groups. Therefore, we recommend standardising CALD indicators and categorisations to guide future studies and enhance understanding of PCa care and outcomes among diverse populations.

### Screening and Early Detection

This review found that most included papers (*n* = 37) provided data on PCa screening and early detection. Findings indicated that one or more subpopulations from CALD backgrounds received PCa screening and early detection less frequently than their non-CALD counterparts. This finding aligns with the well-documented challenges associated with healthcare delivery in the case of cultural and linguistic diversity [13, 92]. Consequently, gaps in screening and early detection for PCa may contribute to worse health outcomes, including more advanced stages of disease at diagnosis. For instance, CALD men (e.g., Asians and Hispanics) presented with a high-risk or metastatic PCa at initial diagnosis more often than non-CALD men [57, 63, 73, 75, 90]. Barriers such as age, socioeconomic and financial constraints, preferred language, and lack of health insurance coverage were identified as factors limiting access to PCa screening and early detection among CALD men. These findings reinforce the importance of considering intersectionality and social determinants of health in men from CALD backgrounds, as these factors can create overlapping and interdependent disadvantages [93].

Our review also found that spending more time in the country of immigration increased the likelihood of getting PSA tested [21, 62]. Evidence from a prior longitudinal study conducted among immigrants highlighted that the more years spent in a host country, the greater the PCa risk due to increased exposure to risk factors [94]. Some attributed risk factors include unhealthy living conditions, reversal of healthy migrant effects, aging, and acculturation, which tend to increase susceptibility and the need for more PCa screening [94]. On the other hand, a longer stay in the host country may indicate enhanced familiarity with the healthcare system, enabling better navigation and optimisation of PCa care [27].

### Screening Practices

Our review identified guidelines-discordant PCa screening practices in CALD men, including PSA testing performed without the men’s awareness and shared decision-making [47]. This result highlights a lack of culturally and linguistically appropriate PCa information, poor patient-physician communication, and a lack of men’s empowerment [95]. Further research is needed to evaluate the power dynamic between CALD men and clinicians and the extent of CALD men’s autonomy in medical decision-making. Both local and international guidelines for PCa emphasise the importance of patient empowerment in healthcare decision-making [31, 96, 97].

Our review also highlighted that men from CALD backgrounds exhibited lower compliance with regular or annual PCa screening examinations, reflecting less adherence to screening guidelines [38, 46, 54, 59, 68]. This finding suggests that men from CALD backgrounds are experiencing barriers to accessing PSA testing. Because no single PSA test can rule out the lifetime risk of PCa, annual PSA/DRE monitoring is crucial.

In half of the reviewed papers on screening and early detection (*n* = 18), men, including those from CALD backgrounds recruited for PCa screening, were under 50. This finding suggests variability in PCa screening practices and inconsistencies in adherence to the US Preventive Services Taskforce guideline [98]. According to this guideline, average-risk men should begin discussions about PSA testing at age 50 or older, considering risk factors, potential benefits, and associated harms. However, our review found that the underlying factors influencing the trade-off between early screening at a younger age and its implications for overdiagnosis and overtreatment remain poorly understood. Additional research is essential to elucidate the effects of early-age PCa screening on unnecessary biopsies, overdiagnosis, and overtreatment in men from CALD backgrounds.

### Barriers to Screening and Early Detection

Language barriers within the CALD population were found to negatively impact the uptake of PCa screening. Men who preferred to speak languages other than English or lacked proficiency in spoken English were less likely to undergo PCa screening than those who did [19–21, 85]. These results are consistent with a growing body of evidence showing that limited language proficiency is associated with low health literacy, poor quality healthcare use, and worse health outcomes [13]. Disparities of PCa care based on preferred spoken language reflect the existence of structural discrimination within the healthcare system. It indicates the system is unresponsive and fails to adequately address the needs and preferences of multicultural populations, including non-English Speaking individuals.

The Healthy Plan 2030 framework, set by the US Department of Health and Human Services, considered people with limited English proficiency to be a priority group for addressing inequities and social determinants of health [99]. Similarly, the Australian National Cancer Control Plan identified and prioritised the CALD people for equity interventions aimed at improving care experiences and health outcomes. The cancer plan suggested strategies such as incorporating multicultural and multilingual workforce in cancer training and care teams is essential for addressing disparities [100]. This approach aims to enhance representation, cultural competency, and patient-centred care and mitigate discrimination and bias by promoting targeted outreach and information provision to disadvantaged groups such as the CALD population.

Other challenges to PCa screening in CALD men may be related to knowledge and attitude-related issues, including lack of knowledge, fear of diagnosis, health-seeking behaviour, preference for Eastern medicine, misconceptions, and a lack of screening habits [26, 28, 49, 52, 53, 70, 83]. This implies cultural, linguistic, and literacy challenges are influencing CALD men’s access to and use of healthcare, resulting in suboptimal health outcomes and experiences. Furthermore, these findings highlight the need for culturally and linguistically sensitive health education to address misconceptions about PCa screening and treatment. Potential interventions include providing translated health education and behavioural change materials. Such interventions are important, given that 64% of men from CALD backgrounds in Australia reported unmet needs for PCa health information in a language other than English [86]. Additional evidence suggests that interventions such as patient navigation programs effectively address the challenge of cultural and linguistic diversity on access to cancer preventive services [101]. Moreover, our scoping review also emphasised the need to sensitise healthcare workers, particularly general practitioners (GPs), regarding the disparities in PSA testing among men from CALD backgrounds.

### Prostate Cancer Treatment

Only twelve papers reported data on PCa treatment, with inconsistent results. Findings underscored that men from CALD backgrounds had less access to PCa treatment, experienced unwarranted treatment variations, and received suboptimal care and non-adherence to AS follow-ups. Although surgery and radiation therapy demonstrate equivalent efficacy for men diagnosed with localised PCa, foreign-born men with intermediate-risk PCa are more likely to receive surgical treatment compared to native-born men. Evidence showed that male patients perceived that surgery may provide complete cancer eradication compared to other treatment modalities; this could shaped by men’s culture, attitude, and preferences [102].

Our review also indicated that CALD patients, particularly refugees, received suboptimal radiation therapy, with a higher non-compliance rate to the prescribed radiation doses. Poor access to standard treatment and a greater non-compliance rate to PCa treatment could be exacerbated by communication barriers and limited healthcare access among disadvantaged groups, including refugees. This highlights the need to improve access to high-quality PCa treatment for humanitarian settlers. In addition, CALD men had higher odds of non-adherence to AS follow-up; this result suggests the need to enhance patient-physician communication, discussion, and patient-centred approaches for improving adherence and engagement [103].

Only two retrospective studies from the US and Norway reported types of surgical procedures performed, including robot-assisted prostatectomy (RAP) [57, 87]. Minimally invasive surgeries such as RAP have implications for immediate patient outcomes in terms of lower rates of blood loss, shorter hospital stays, and less pain [104]. Results from earlier research indicated that RAP was performed less frequently on African Americans and patients without private health insurance than on white patients and those with health insurance [105]. In our review, there is a marked lack of evidence regarding the management of metastatic PCa, such as the utilisation of treatment intensification for the treatment of hormone-sensitive metastatic PCa. Building this evidence base could one day facilitate understanding regarding translating clinical trials into real-world patient management for men from CALD backgrounds [106].

### Follow-up, Supportive, and Palliative Care

None of the studies in our review assessed palliative or end-of-life care among men from CALD backgrounds. Older age and advanced-stage PCa at diagnosis may increase the need for palliative and end-of-life care, which needs to be supported with quality evidence [14]. Prior research in Canada reported that cultural contexts had influenced end-of-life care preferences among Asian immigrants; this can include misconceptions and attitudes toward death and hospice care [107]. Only five of the studies included in our review presented evidence regarding PCa follow-up and supportive care in men from CALD backgrounds diagnosed with PCa. Studies have shown that PCa survivors of CALD men had unmet needs for emotional, mental, social, and psychological support [71, 84, 89]. Men diagnosed with PCa sought informal, supportive care from family members or community-based organisations and desired integration of cultural and traditional medicine into their care. The finding can suggest CALD men demand formal and standardised follow-up care tailored to their cultural and linguistic preferences and diverse needs. In addition, studies have shown men from CALD backgrounds experienced a fractured transition of care between the procedure-focused healthcare system and self-management practices associated with follow-up care. Although telehealth follow-up care can resolve logistical challenges, it has faced acceptability issues by PCa survivors due to uncertainties that may affect PCa survivors’ and clinicians' interactions [89]. Therefore, further research is required to design supportive care and survivorship services that address the specific needs of men from CALD backgrounds and build self-management skills tailored to their cultural and linguistic contexts.

### Treatment Outcomes

Only four papers have examined PCa treatment outcomes, yielding inconsistent results. CALD men from Hispanic backgrounds living in high- and low-ethnic enclaves demonstrated better PCa-specific survival compared to US-born men [40]; however, Puerto Ricans had worse PCa-specific mortality than non-Hispanic Black men (NHB)[77]. These conflicting findings underscore the need for additional research, including meta-analyses, to better understand these disparities and suggest tailored interventions aimed at improving outcomes for CALD men.

### Limitations of the Study

Our study has some limitations. First, we only considered papers published in the English language. Second, our review operationally defined CALD backgrounds within the context of migrant flows from non-English-speaking and non-Western countries to mainly Anglophone countries. Third, there is considerable heterogeneity among males from CALD backgrounds, and, indeed—no international standard definition for this group. Only a small number of CALD population subgroups were examined in our review. Despite adhering to rigorous methodological approaches to conduct this review, potential subjectivities in study selection may limit the generalisability of our findings.

## Conclusions

The findings from this review indicated inequity in PCa screening, with men from CALD backgrounds undergoing PCa screening less frequently than their non-CALD counterparts. Patients from CALD backgrounds had less access to PCa treatment, unwarranted treatment variations and suboptimal treatment, and mortality was worse in some CALD population subgroups. This scoping review suggests that future research needs to extend beyond PCa screening and early detection to later steps of the PCa care pathway. Future research should also consider using longitudinal study designs to examine the impact of provider, health system, and policy-level factors on PCa management, follow-up, and end-of-life care in men from CALD backgrounds.

## Key References

- Stone BV, et al. The effect of limited english proficiency on prostate-specific antigen screening in American men. World J Urol. 2024;42(1):54.
  - ○ This study highlighted that limited English language proficiency influenced prostate cancer-specific antigen testing utilisations in the United States. It helps the understanding of poor patient-physician communication and language difficulty are barriers to screening and early detection of PCa, particularly important evidence for the migrant population.
- Persaud H, et al. Barriers to prostate cancer screening among indo-guyanese. J Community Health. 2021;46:591–6.
  - ○ This study investigated factors affecting participation in prostate cancer screening among immigrants in the United States. Lack of knowledge and fear of diagnosis for prostate cancer, together with cultural factors like refusal of rectal examination, can impede participation in prostate cancer screening. This can provide insights into the critical importance of designing culturally and linguistically adapted health education for migrants.
- Estrada-Mendizabal RJ, et al. Prostate cancer disparities in metastatic and treatment status for Hispanic Americans based on country of origin compared to Non-Hispanic whites using the national cancer database. Clin Genitourin Cancer. 2024;22(1):e148–e155.e1.
  - ○ This study highlights that Hispanics, defined by country/regions of origin, had higher odds of presentation with initial metastatic prostate cancer but had less access to treatment compared to non-Hispanic Whites. This can highlight the importance of considering patients with the country of birth, which is a proxy indicator of language, culture, and practices.
- Heard JR, et al. Definitive treatment choice among black immigrants with prostate cancer: analysis of patient surveys distributed at a single safety-net institution. Prostate. 2022;82(13):1258–63.
  - ○ This study describes prostate cancer treatment variations by patients backgrounds. The result highlights that foreign-born patients are more likely to opt for surgical treatment of prostate cancer vs radiation therapy compared to the US-born. However, as years lived in the country of destination increased, the rate of radiation therapy for intermediate-risk PCa also increased.
- Jain B, et al. Prostate Cancer Disparities in Risk Group at Presentation and Access to Treatment for Asian Americans, Native Hawaiians, and Pacific Islanders: A Study With Disaggregated Ethnic Groups. JCO Oncol Pract. 2022;18(2): E204–E218.
  - ○ This study examines clinical characteristics at presentation and access to treatment among ethnic Asian groups in the United States. The result highlighted there were differences in high-risk prostate cancer at presentation and access to treatment between Asian Pacific Islanders and Whites. In addition, significant differences were highlighted among the same ethnic groups.
- Johnson JA, et al. Associations of prostate-specific antigen (PSA) testing in the US population: results from a national cross-sectional survey. J Community Health. 2021;46:389–98.
  - ○ This study examined the association between nativity status and prostate-specific antigen testing. Foerign-born men were less likely to undergo prostate-specific antigen testing compared to native-born. The study can help our understanding of the importance of addressing inequities in the immigrant population.
- Li Y, et al. Gains in insurance coverage following the affordable care act and change in preventive services use among non-elderly US immigrants. Preventive Med. 2021;148:106546.
  - ○ This study highlights how the 2014 Affordable Care Act reform in the US improved the utilisation of preventive health services, including PSA testing, among newly insured foreign-born adults. The study addressed policy-level factors influencing the use of preventive health services.
- Swami N, et al. Localized prostate cancer disparities in risk group at presentation and access to treatment for Hispanic men. Prostate Cancer Prostatic Dis. 2023;26(2):309–16.
  - ○ This study describes prostate cancer presentation at diagnosis and access to treatment by country of birth. Hispanic patients had higher odds of presentation with high-risk localised prostate cancer and experienced less access to treatment than non-Hispanic Whites.
- Eren MF, et al. Radiation therapy for prostate cancer in Syrian refugees: facing the need for change. Front Public Health. 2023;11:1172864.
  - ○ This paper describes the experience of Syrian refugees with prostate cancer patients in Turkey. The results revealed that despite having advanced disease at diagnosis, patients had less access to treatment and had greater non-compliance to prescribed therapies.

## Supplementary Information

Below is the link to the electronic supplementary material.

Supplementary file1 (DOCX 15 KB)

Supplementary file2 (DOCX 65 KB)

## Data Availability

No datasets were generated or analysed during the current study.
