# Supplementary material for: Patterns of Care and Treatment Outcomes Among Men Diagnosed with Prostate Cancer from Culturally and Linguistically Diverse Backgrounds: A Scoping Review
Source: Curr Oncol Rep. 2025 Mar 29;27(5):552–71. doi: 10.1007/s11912-025-01660-8 (PMC12081574; doi:10.1007/s11912-025-01660-8)
Supplement: Supplementary file 1 — Supplementary file1 (DOCX 15 KB) [file 11912_2025_1660_MOESM1_ESM.docx]

Supplementary file 1: MEDLINE search strategy

| # | Searches |
| --- | --- |
| 1 | Prostatic Neoplasms/ |
| 2 | prostat* cancer*.mp. |
| 3 | prostatic neoplasms.mp. |
| 4 | (Prostat* adj3 survivor*).mp. |
| 5 | (Localized adj2 Prostate Cancer).mp. |
| 6 | Or/1-5 |
| 7 | Minority Groups/ |
| 8 | Population Groups/ |
| 9 | Cultural Diversity/ |
| 10 | Cultural Characteristics/ |
| 11 | Culture/ |
| 12 | cross-cultural comparison/ |
| 13 | (((cultur* or lingustic*) adj3 divers*) or CALD).mp. |
| 14 | (ethnocultural adj2 (differen* or variation* or divers*)).mp. |
| 15 | (migrant* or immigrant* or emigrant*).mp. |
| 16 | exp "Emigrants and Immigrants"/ |
| 17 | multilingualism/ |
| 18 | Bilingualism.mp. |
| 19 | (english adj3 language).mp |
| 20 | limited english proficiency/ |
| 21 | communication barriers/ |
| 22 | Non-English Speaking.mp. |
| 23 | (Country adj2 birth).mp. |
| 24 | (foreign-born or overseas born).mp. |
| 25 | "first generation immigrant".mp. |
| 26 | "second generation immigrant".mp. |
| 27 | ethnic group.mp. |
| 28 | exp Ethnicity/ |
| 29 | Or/7-28 |
| 30 | 6 AND 29 |
