# Supplementary material for: Patterns of Care and Treatment Outcomes Among Men Diagnosed with Prostate Cancer from Culturally and Linguistically Diverse Backgrounds: A Scoping Review
Source: Curr Oncol Rep. 2025 Mar 29;27(5):552–71. doi: 10.1007/s11912-025-01660-8 (PMC12081574; doi:10.1007/s11912-025-01660-8)
Supplement: Supplementary file 2 — Supplementary file2 (DOCX 65 KB) [file 11912_2025_1660_MOESM2_ESM.docx]

Supplementary Table 1: Overview of included studies (n=58)

| Author(s), publication year,  Country | | Study overview | Study design | Data collection methods, and year(s) of data collection | Data analysis and confounding factors adjusted | CALD-defining characteristics | CALD population characteristics (percent of CALD and non-CALD population) | Total sample size | Age (years)  Mean/Median/Range | |
| --- | --- | --- | --- | --- | --- | --- | --- | --- | --- | --- |
|  |  |  |  |  |  |  |  |  | Total population | Specific to CALD |
| Anderson et al., ^[49]^ ^a^,  UK | 2013 | PCa among African and Caribbean men | Qualitative | In-depth interviews, year of data collection not reported | Thematic analysis | Ethnicity, self-identified | CALD: African and Afro-Caribbean (100%) | 7 | NA | Range:  60-76 |
| Bamidele and McCaughan ^[79] d^,  UK | 2022 | Exploring PCa treatment decision-making process | Constructivist grounded theory | Face-to-face, telephone, and Skype interviews, 2016 to 2018 | Thematic analysis | Ethnic origin, self-identified | CALD: Black Caribbean or Black African (100%) | 25 | NA | Mean:  65 |
| Chu et al. ^[51]^ ^b^,  US | 2015 | Effect of providers’ experience on PCa treatment choices | Cohort | Self-administered questionnaire and document review, 2011 to 2013 | Binary logistic regression  Adjusted for age, years in KPSC, number of robotic operations, and physician fellowship training | Preferred language, self-identified | CALD: Spanish (0.7%) and other language-speaking backgrounds (10.5%)  Non-CALD: English-speaking backgrounds (88.8%) | 713 | Mean (SD):  61.8 (7.29) | NR |
| Cobran et al. ^[52]^ ^a^  US | 2018 | Factors influencing PCa screening | Mixed methods | Pilot study survey, face-to-face interview, and focus group discussion, 2011 to 2012 | Thematic analysis | Country of birth, self-identified | CALD: Caribbean-born (54.8%)  Non-CALD: African-American (45.2%) | 31 | Range:  39-75 | NR |
| Cobran et al. ^[24] a^,  US | 2014 | PCa fatalism perceptions and screening behaviour | Cross-sectional community-based | self-administered survey, 2012 | Binary logistic regression  Adjusted for age, general health, income, education, marital status, nativity, spirituality, acculturation, insurance, physician recommendation, regular physician, PCa fatalism, level of PCa knowledge | Nativity, self-identified | CALD: Caribbean-born blacks (44.5%)  Non-CALD: US-born blacks (55.5%) | 211 | Range:  39-75 | NR |
| Conde et al. ^[53]^ ^a^,  US | 2011 | Culturally relevant interventions are important for PCa screening | Exploratory qualitative | Face-to-face interviews and focus group discussion, year of data collection NR | Content analysis | Ethnicity, self-identified | CALD: Filipino (100%) | 20 | NA | Mean (SD):  56 (8) |
| Consedine et al.^[54] a^,  US | 2009 | PCa screening adherence among ethnic groups | Cross-sectional | Face-to-face interviews, 2004 to 2006 | Multinomial logistic regression  Adjusted for age, income, education, relationship, ethnicity, structural and disease history characteristics | Immigration status, self-identified | CALD: Immigrant Jamaicans and Trinidadian and Tobagonians (50.6%)  Non-CALD: European-American and African American (49.4%) | 533 | Mean (SD):  AA: 54.30 (7.29)  EA: 58.20 (8.47) | Mean (SD),  Jamaicans: 53.18 (6.68), Trinidadians and Tobagonians: 52.78 (6.40) |
| Dallo and Kindratt ^[55] a^  US | 2015 | Preventive health service uptake by country of birth | Cross-sectional | Survey, 2000 to 2011 | Binary logistic regression  Adjusted for age, education, poverty ratio, employment, insurance, smoking, BMI, years in US, place often received healthcare | Nativity, self-identified | CALD: Foreign-born (Europe and Arab nations) (3.1%)  Non-CALD: US-born Whites (96.9%) | 91,636 | Mean (SE)  US-born:  46.6 (0.10) | Mean (SE),  Europe-born: 49.10 (0.42)  Arab-born: 38.60 (1.07) |
| Dobbs et al. ^[56]^  ^d^  US | 2018 | PCa disparities in Hispanics' country of birth | Retrospective cohort | Population-based cancer registry (SEER) | Age, years of diagnosis, PSA, treatment, tumour stage, high school attainment, poverty, unemployment, and income | Country of birth | CALD: Hispanics (100%) | 72,134 | Median (IQR): 67 (60-74) | NA |
| Eren M.F et al. ^[90] b^  Turkey | 2023 | Utilisation of radiation therapy for PCa | Retrospective cohort | Cancer centres databases treated with radiation therapy 2015 to 2019 | Descriptive analysis used to summarise results. | Refugee/immigration status | CALD: Syrian refugee (100%) | 137 | NA | Median (IQR): 65 (54-86) |
| Estrada-Mendizabal et al. ^[57] b^  US | 2023 | Characterise clinical differences in PCa metastatic presentation and Rx status among Hispanics | Retrospective cohort | National Cancer Database 2010 to 2016 | Multivariable logistic regression  Adjusted for sociodemographic and clinical data. | Country of birth | CALD: Hispanics (Mexican, Puerto Rican, Cuban, and south and central America (1.3%)  Non-CALD: Non-Hispanic Whites (98.7%) | 428,829 | Median (IQR): 65 (59-71) | Median (IQR), Mexican: 65 (59-70), Puerto Rican, 65 (60-70), Cuban, 68 (62-73), and South and Central America, 64 (58-71) |
| Fedewa et al . ^[58] b^,  US | 2013 | PCa disease severity and country of origin | Follow-up | Population-based cancer registry (SEER), 2004 to 2009 | Descriptive with chi-squared test | Country of birth,  administrative data | CALD: Jamaican-born Black (0.2%) and West African-Born (0.2%)  Non-CALD: US-Born Black (18%) & Whites (81.6%) | 110,048 | Mean SD:  US-born black: 68.2 (9.87),  US-born black: 65.1 (9.82) | Mean (SD),  West African-born lack: 61.2 (8.88),  Jamaican-born black: 64.40 (9.70) |
| Glenn et al. ^[59] a^,  US | 2009 | The rate of PCa screening among South Asians | Cross-sectional, community-based | Face-to-face and telephone interviews, 2004 to 2005 | Binary logistic regression  Adjusted for ethnicity, age, education, income, insurance, years in US | Ethnicity, self-identified | CALD: South Asians (Bangladeshi, Indian, Pakistani, Sri Lankan, Nepali, and other) (100%) | 344 | NA | Range:  18+ |
| Gupta et al. ^[60] a^,  US | 2014 | Association between time to treatment and PSA changes | Follow-up | Facility-based patient’s medical record review, 2005 to 2013 | ANOVA  No adjustment for confounders | Preferred language, medical records | CALD: Spanish (10.2%), Haitian Creole (10.2%), and other languages other than English (6.8%)  Non-CALD: English Speaking (72.8%) | 147 | Median (IQR):  67 (7.1) | NR |
| Heard et al. ^[61]^ ^b^,  US | 2022 | Exploring treatment choices among immigrants | Cross-sectional | Survey and medical record review, 2019 to 2020 | Binary logistic regression  Adjusted for age | Country of birth, self-reported | CALD: Foreign-born (45.5%)  CALD: US-born (54.5%) | 253 | Range:  45-82 | NR |
| Hei et al. ^[62] a^,  US | 2019 | Neighbourhood cohesion and cancer screening | Cross-sectional, community-based | Population-based survey (PINE project), 2011 to 2013 | Binary logistic regression  Adjusted for age, education, income, country of origin, marital status, number of children, living arrangement, years in US, preferred language, self-reported health status, | Country of birth, self-identified | CALD: Self-identified Chinese Americans who cannot read and speak English (100%) | 3,159 | NA | Mean (SD):  72.8 (8.3) |
| Ilunga et al. ^[25]^ ^a^,  US | 2016 | Colorectal and prostate cancer screening by Nativity | Cross-sectional, community-based | Population-based survey, 2015 | Binary logistic regression  Adjusted for sociodemographic, clinical and behavioural characteristics | Nativity, self-identified | CALD: Foreign-born (NR)  Non-CALD: US-born (NR) | 5,651 | Mean (SD):  59.7 (12.10) | NR |
| Jain et al. ^[63]^ ^b^,  US | 2022 | Patterns of PCa treatment by ethnic group | Retrospective cohort | National cancer database, 2004 to 2017 | Ordinal logistic regression  Adjusted for age, race/ethnicity, risk group, facility type, median household income | Ethnicity, clinical registry | CALD: Self-identified as Thai, White, Asian Indian, Chinese, Vietnamese, Korean, Japanese, Filipino, Laotian, Pakistani, Kampuchean, and Hmong (1.7%)  Non-CALD: Others (Native Hawaiian, etc) (98.3%) | 980,889 | Mean (SD):  66.0 (60.7)  Range: 18+ | NR |
| Johnson et al. ^[64] a,^  US | 2020 | PSA testing among foreign-born and US-born population | Cross-sectional, community-based | Population-based survey (NHIS) data, 2010 to 2015 | Binary logistic regression  Age, race/ethnicity, education, marital status, family history of cancer, insurance, nativity, and region of birth | Nativity, self-identified | CALD: Foreign-born (17.7%) from Latin America, Europe/Russia, Africa/Middle East, Asia/India, and elsewhere  Non-CALD: US-born natives (82.3%) | 15,372 | Range:  40-85 | NR |
| Kleier ^[65]^ ^a^,  US | 2020 | Perceived susceptibility increased PCa examinations | Cross-sectional, community-based | A self-administered questionnaire, year of data collection NR | Binary logistic regression, unadjusted | Preferred language and immigration history, self-identified | CALD: Haitian-American (100%) | 143 | NA | Range:  46-70 |
| Kudadjie et al. ^[66]^ ^a^,  US | 2008 | Ethnic disparities in PCa screening | Cross-sectional | Face-to-face interviews study, year of data collection not reported | Linear regression  Adjusted variables are not clear | Ethnicity, self-identified | CALD: Hispanic descent—Dominicans, African descent—Haitians, white immigrant group—men from Eastern Europe (Russia, Ukraine, Belarus), Caribbean (89.6%)  Non-CALD: European-American and African American (10.4%) | 308 | Mean (SD):  58.60 (5.9) | Mean (SD), EC: 58.6(5.6), HA: 58.0(6.1), Dom: 57.6 (5.3), EE: 61.4 (6.3) |
| Kum et al. ^[81]^ ^b^,    UK | 2021 | Adherence and outcomes of PCa among men on active surveillance | Retrospective follow-up | Hospital database, 2005 to 2016 | Binary logistic regression and Cox-proportional hazards regression  Adjusted for age, calendar year, and Gleason score | Ethnic origin, patients’ records | CALD: Afro-Caribbean (22.5%)  Non-CALD: Whites (77.5%) | 458 | Median (IQR):  63 (58-68) | Median (IQR):  63 (54-68) |
| Lee and Jung ^[26] a^,  US | 2013 | The role of culture to PCa screening | Cross-sectional Community-based | Face-to-face interviews and self-administered questionnaire, 2009 | Binary logistic regression  Adjusted for age, marital status, education, income, and employment, | Immigration status, self-report | CALD: Korean American immigrants (100%) | 134 | NA | Mean (SD):  67.03 (8.67) |
| Lee et al. ^[46]^ ^a^,  US | 2012 | Patterns of DRE and associated factors | Cross-sectional | Face-to-face interviews, 2004 to 2006 | Multinomial regression analysis  Adjusted variables not listed | Country of birth, self-identified | CALD: Jamaican, and Trinidadian/Tobagonian (50.6%)  Non-CALD: US-born African American and Whites (49.4%) | 533 | Rage:  45-70 | NR |
| Lee et al.  ^[38]^ ^a^,  US | 2011 | Frequency of PSA testing among Jamaican and Trinidad/Tobagonian men | Cross-sectional | Face-to-face interviews, 2004 to 2006 | Binary and multinomial logistic regression  Adjusted for age, income, insurance, education, and family history of cancer | Ethnicity, self-identified | CALD: Jamaican- (27.6%) and Trinidad/Tobagonian-born (23.1%)  Non-CALD: US-born African American (26.6%) and Whites (22.7%) | 533 | Mean (SD):  54.5 (7.5) | NR |
| Lepore et al. ^[47]^ ^a^,  US | 2017 | Undisclosed PCa screening among immigrants | Randomised control trial | Telephone interviews and medical record review, 2005 to 2006 | Binary logistic regression  Adjusted for intervention | Immigration status, healthcare register | CALD: Predominantly black immigrants (100%) | 142 | NA | Range:  45-70 |
| Lepore et al. ^[48]^ ^a^,  US | 2012 | Effect of decision support intervention among immigrants | RCT, Community-based | Telephone interviews (pre and post intervention), 2005 to 2006 | Analysis of Covariance (ANCOVA)  Adjusted for education, Prior PSA claim, state of anxiety | Immigration status, healthcare register | CALD: Black immigrants, predominantly of Caribbean origin (100%) | 490 | NA | Mean (SD):  55.04 (6.29) |
| Li et al. ^[67] a^,  US | 2021 | Effect of health insurance gain on preventive service use | Retrospective cohort | Secondary data source (MEPS), 2010 to 2016 | Generalised estimating equation  Adjusted for race/ethnicity, gender, age, education, family income, employment, region, self-reported general health, self-reported mental health | Nativity,  Administrative data (Medicare) | CALD: Foreign-born Hispanic, non-Hispanic Asia (31.9%)  Non-CALD: US-Born, Non-Hispanic White, Non-Hispanic Blacks, Non-Hispanic Others (68.1%) | 22,606 | US-born,  Mean (SD):  44.70 (11.11) | Mean (SD):  43.38 (10.23) |
| Loeb et al. ^[88] a^,  Sweden | 2013 | PCa risk group and country of birth | Case-control | Secondary data source (PCa database in Sweden: PCBaSe), 1991 to 2008 | Binary logistic regression and stratified analysis by years since migration status, duration of residence, stage of the disease  Adjusted variables not mentioned | Immigration status, administrative data | CALD: Immigrants from Africa, America, Europe, Asia, the Middle East, and Latin America; cases (1.2%) & controls (8.4%)  Non-CALD: Sweden-born Cases (16.0%) & controls (74.4%) | 679,972 | Median (IQR):  72 (65-78) | Median (IQR):  69 (63-75) |
| Ma et al. ^[69]^ ^a^,  US | 2009 | PCa screening among Asian Americans | Cross-sectional | Self-administered questionnaire, 2005 to 2006 | Descriptive statistics only | Nationality (ethnicity), self-identified | CALD: Ethnic groups (Chinese, Korean, Vietnamese, Cambodian) (100%) | 2,011 | NA | Range:  18+ |
| Ma et al. ^[68]^ ^a^,  US | 2012 | Sociocultural health behaviour model and PCa screening | Cross-sectional | Self-administered questionnaire, 2006 | Structural equation modelling | Nationality and preferred language, self-identified | CALD: Self-identified Chinese American (100%) | 163 | NA | Mean:  60.2 |
| Magin et al. ^[19] a^,  Australia | 2017 | Non-symptomatic PSA ordering practice | Cross-sectional, facility-based | Face-to-face interviews and secondary data review, 2010 to 2014 | Generalised Estimating Equation  Adjusted for age, NESB, practice status, term, and provider characteristics | Language spoken at home, self-identified | CALD: Non-English-speaking backgrounds (6.5%)  Non-CALD: English-speaking backgrounds (93.5%) | 856 | Range:  40+ | NR |
| Malika et al. ^[70] a^,  US | 2021 | PCa knowledge and screening among blacks | Mixed methods | Mixed methods study, interviews (key informant, focused group discussion, and survey) | Binary logistic regression  Adjusted for age, income, ethnicity. Thematic analysis of qualitative component | Immigration status, self-identified | CALD: Caribbean and African immigrants (56.8%)  Non-CALD: African Americans (43.2%) | 396 | Mean (SD),  AA: 59.34 (9.07) | Mean (SD),  CI: 58.78 (8.58), AI: 56.23 (8.04) |
| Martins et al.  ^[80]^ ^a^,  US | 2021 | Ethnic difference in primary care use for PCa | Mixed methods | Face-to-face interviews, and survey, 2016 to 2017 | Binary logistic regression analysis  Adjusted for age, comorbidity, and presence of LUTS. Thematic analysis of qualitative component | Ethnicity, self-identified | CALD: Asian and Blacks (32.4%)  Non-CALD: White men (67.6%) | 274 | Median (IQR):  67 (58-74) | Median (IQR),  Asian: 63 (56-74),  Black: 65 (57-73) |
| McGuiness et al. ^[85] a^,  Australia | 2017 | Need for cognition predicted and self-reported DRE | RCT | Self-administered questionnaire, 2013 | Structural equation modelling | Language spoken at home, self-identified | CALD: Speak a language other than English at home (16.4%)  Non-CALD: Speak English at home (83.7%) | 585 | Mean (SD):  61.4 (6.7) | NR |
| Moreno et al. ^[27]^ ^a^,  US | 2019 | Fatalism and adherence to cancer screening among Hispanic/Latino | Cross-sectional | Self-administered questionnaire, 2008 to 2011 | Binary logistic regression  Adjusted for age, income, insurance, education, Hispanic backgrounds | Ethnicity, self-identified | CALD: Hispanic/Latino backgrounds (100%) | 5,313 | NA | Mean (SD):  59.33 (0.33) |
| Nair-Shalliker et al. ^[44]^ ^a^,  Australia | 2018 | Determinants of PSA testing | Cross-sectional | Self-administered questionnaire, 2006–2009 | Binary logistic regression  Adjusted for age, place of residence, qualification, and income | Country of birth, linked data | CALD: Foreign-born (26.3%)  Non-CALD: Australian-Born (73.7%) | 62,765 | NA | Range:  45+ |
| Nanton and Dale ^[84]^ ^d^,  UK | 2011 | The role of ethnicity in PCa experiences | Qualitative | Face-to-face interviews, year of data collection not reported | Thematic analysis | Country of birth, self-identified | CALD: First-generation African-Caribbean immigrants (Jamaican-born) (100%) | 16 | NA | Median:  72.5 |
| Odedina et al. ^[72] a^,  US | 2011 | Perceived behavioural control and severity and PCa screening | Cross-sectional, community-based | self-administered survey, 2008 to 2009 | Descriptive with chi-squared test | Ethnicity, self-identified | CALD: African-born blacks (9.8%) and Caribbean-born blacks (10.3%)  Non-CALD: Native-born blacks (79.9%) | 2,864 | Range:  40-70 | NR |
| Odedina et al. ^[39] a^,  US | 2011 | PCa risk reduction and early detection practices | Cross-sectional, community-based | Self-administered questionnaire, 2008 to 2009 | Descriptive with chi-squared test | Country of birth, self-identified | CALD: African-born blacks (10.4%) and Caribbean-born blacks (10.5%)  Non-CALD: Native-born blacks (79.1%) | 3,040 | Range,  40-70 | NR |
| Odedina et al. ^[71] d^,  US | 2017 | Experiences and needs of PCa survivors | Qualitative | Community-based, in-depth interview, 2006 to 2010 | Dimensional analysis | Nativity, self-identified | CALD: Caribbean-born blacks (45.2%)  Non-CALD: Native-born blacks (54.8%) | 31 | Range,  50+ | NR |
| Patel et al. ^[73] b^,  US | 2013 | Pathological characteristics PCa among South Asians | Retrospective follow-up facility-based | Facility-based document review, 1990 to 2011 | Descriptive with chi-squared test | Nativity, medical records | CALD: South Asians (0.9%)  Non-CALD: African American (99.1%) | 10,053 | Mean (SD), AA: 59.0 (7.8) | Mean (SD),  61.40 (6.90) |
| Persaud et al. ^[28] a^,  US | 2020 | Barriers to DRE among immigrants | Qualitative | Community-based face-to-face interview, data collection period not reported | Thematic analysis | Immigration status, self-identified | CALD: Asian Indian, Indo-Guyanese immigrants (100%) | 20 | NA | Range:  45-75 |
| Schupp et al. ^[40] c^,  US | 2014 | Survival outcomes of PCa | Follow-up facility based | Follow-up, facility-based, California Cancer registry (CCR), 1995 to 2008 | Cox proportional hazards regression  Adjusted variables NR | Immigration status, administrative data (social security number) | CALD: Foreign-born men (56.2%)  Non-CALD: US-born (43.8%) | 35,427 | NR | NR |
| Seymour et al. ^[83] a^,  UK | 2016 | Perceptions and Psychosocial Aspects of DRE | Qualitative | Face-to-face semi-structured interviews, year of data collection not reported | Synthetic discursive approach | Country of origin, self-identified | CALD: African-Caribbean (Trinidadian and Tobagonian and Jamaican) (100%) | 20 | NA | Range:  59-83 |
| Smigelski et al. ^[74] b^,  US | 2021 | Sociodemographic and neighbourhood factors influencing aggressive local therapies | Ecological | Population-based  database (NYSPACED), 2004 to 2016 | Multi-level mixed effects logistic regression  Adjusted for age, race, year of diagnosis, and borough | Foreign-born & language proficiency, administrative data | CALD: Foreign-born residents’ rate (mean: 37%) and limited English proficiency rate (23%) in Borough  Non-CALD: US-born (NR) | 40,668 | Range:  40-79 | NR |
| Stone, Labban et al. ^[20] a^  US | 2024 | Effect of English language proficiency on PSA screening in American men | Cross-sectional | Secondary data from MEPS collected data from 2013 to 2016 | Multivariable logistic regression Adjusted for age, race, years of survey, income, region, routine visit, and primary care visit | English language proficiency | CALD: Who spoke English not well or not at all (61%)  Non-CALD: Who spoke English very well and well (39%) | 4,835 | NR | Range:  55+ |
| Swami N et al. ^[75] b^  US | 2023 | Localised PCa disparities in presentation and access to treatment | Retrospective cohort | National Cancer Database |  | Country of birth | CALD: Hispanics (Mexican, Cuban, Puerto Rica, Dominican, other not specified Spanish, or  Non-CALD: Non-Hispanic White, Non-Hispanic Black, and non-Hispanic Other | 895,087 | Median (IQR)age NHW: Non-Hispanic white: 65 (59-71), Non-Hispanic Black (63 (58-69), and Non-Hispanic Other 64 (58-70) | Median (IQR): Hispanic white: 65 (59-71), Hispanic Black (63 (58-69), and Hispanic Other 64 (58-70) |
| Thogersen et al. ^[87]^ ^b^,  Norway | 2020 | Time to diagnosis and treatment commencements | Retrospective cohort | Population-based cancer registry, 1990 to 2014 | Cox-proportional hazards regression and binary logistic regression  Adjusted for age, year of diagnosis, place of residence, marital status, personal income, level of education, stage of diagnosis, and comorbidity | Immigration status,  administrative data | CALD: First- and second-generation immigrants (3.7%)  Non-CALD: Norwegians (96.3%) | 251,343 | Mean (SD), Norway: 68.2 (12.3) | Mean (SD),  Western Immigrant:  65.70 (12.60),  Non-Western Immigrant:  56.60 (13.80) |
| Tim Wong  ^[86] a^  Australia | 2014 | Knowledge, attitude and screening practices of PCa | Mixed methods | Self-administered survey and face-to-face interviews, 2014 | Descriptive statistics | Preferred language, country of birth, self-identified | CALD: Arabic, Chinese, Italian, Greek, and Vietnamese (an average of 50 participants from each language group), 100%) | 256 | NA | Range:  40+ |
| Valdovinos et al. ^[76] a^,  US | 2016 | Perceived discrimination and cancer screening adherence | Cross-sectional | Survey data, 2008 to 2011 | Binary logistic regression  Adjusted for sociodemographic, individual-level socioeconomic and behavioural factors | Nativity, administrative data | CALD: Foreign-born in the Caribbean and Latin American (NR)  Non-CALD: US-born (NR) | 16,415 | Range:  50-74 | NR |
| Velasquez et al. ^[77] c^,  US | 2018 | Mortality outcomes after prostatectomy | Cohort | Population-based cancer registry (SEER), 2004 to 2013 | Fine-Gray competing risk regression model  Adjusted for age, disease stage summary, stage, individual-level SES composite score, insurance, marital status, and residence. | Ethnicity, administrative data | CALD: Non-Hispanic Black Hispanic Latino, Asian American, or Pacific Islander (25.1%)  Non-CALD: Non-Hispanic Whites (74.9%) | 180,794 | Mean (SD):  61.2 (7.4) | Mean (SD),  NHB: 58.70 (7.40), Hispanic: 61.20 (7.60),  Asian American /Pacific Islander: 63 (7.10) |
| Wagland et al. ^[82] d^,  UK | 2020 | Life adjustments after PCa treatment | Mixed methods | Telephone interviews, 2015 to 2016 | Thematic analysis[61, 63, 74, 84] | Country of birth, self-identified | CALD: Black African and Black Caribbean (8.8%)  Non-CALD: Whites (91.2%) | 160 | Mean:  66.7 | Median (IQR):  66 (55-85) |
| Wallner et al. ^[50]^ ^a^,  US | 2012 | Rate of PSA testing | Retrospective cohort | Facility-based secondary data source from KPSC medical centre, 1998 to 2007 | Descriptive with chi-squared test | Preferred language, registry | CALD: Preferred language other than English of Spanish (5.0%), Asian languages (0.6%), and other (0.2%)  Non-CALD: English-speaking backgrounds (94.2%) | 2,061,047 | Range:  18+ | NR |
| Weber et al. ^[21]^ ^a^,  Australia | 2009 | Cancer screening among immigrants | Cross-sectional, community-based | Secondary data from the 45 and Up study, 2006 | Binary logistic regression  Adjusted for age, education, family history, and health insurance status | Country of birth, self-identified | CALD: Immigrants from Asia, America, Africa, Europe, the Middle East, and elsewhere in Oceania (21.5%)  Non-CALD: Australia-born (78.5%) | 31,041 | Range:  45+ | NR |
| Weber et al. ^[45] a^,  Australia | 2014 | Cancer screening among immigrants | Cross-sectional | Self-administered population-based survey, 2006 | Binary logistic regression  Adjusted for age, education, family history, and health insurance status | Immigration status, administrative data | CALD: Immigrants (25.6%)  Non-CALD: Australian-born (74.4%) | 189,917 | Mean (SD):  64.7 (10.8) | Mean (SD):  65.80 (10.80) |
| Xu et al.  ^[78] b^,  US | 2015 | Clinical and prognostic factors of PCa among US- and Foreign-born Asians | Retrospective follow-up | Population-based registry (SEER), 2004 and 2009 | Descriptive with chi-squared test | Nativity,  SEER registry | CALD: Foreign-born Asians (Chinese, Filippo, and Japanese) (69.4%)  Non-CALD: US-Born Asians (30.6%) | 7,824 | Mean (SD):  71.0 (9.51) | Mean (SD):  68.50 (8.98) |
| Young K et al. ^[89]c^  Canada | 2023 | Care experiences of Chinese Canadian survivors | Phenomenological exploratory-descriptive qualitative study | Semi-structured interview | Thematic analysis | Ethnicity, self-identified | CALD: self-identified Chinese or Chinese Canadian (100%) | 14 | Mean (SD): 66 (8.43) | NA |
| Abbreviations: AA/PI: Asian Americans/ Pacific Islander, CALD: Culturally and linguistically diverse, CCR: California Cancer Registry, IQR: Interquartile range, JBB: Jamaican-Born Blacks, KPSC: Kaiser Permanente Southern California, LUTS: Lower Urinary Tract Information, MEPS: Medical Expenditure Panel Survey, NA: Not applicable, NHIS: National Health Interview Survey, NR: Not reported, NYSPACED: New York State Public Access Cancer Epidemiology Data, PCa: Prostate Cancer, PCBaSe: Prostate Cancer Database Sweden, PINE: Population Study of Chinese Elderly, SD: Standard deviation, SEER: Surveillance Epidemiology and End Results, UK: United Kingdom. a, b, c, and d denote studies focused on PCa screening, treatment, outcomes, follow-up, and supportive care, respectively. | | | | | | | | | | |

Supplementary Table 2: Summary of studies with statistical estimates and differences in prostate cancer screening and early detection (n=31 studies)

| Author (s), publication year, country | | Key findings | Brief summary of statistics |
| --- | --- | --- | --- |
| Weber et al. ^[45]^  Australia | 2014 | In Australia, immigrants from regional areas were more likely to undergo PSA testing. | Immigrants from the regional area were more likely to undergo PSA testing than their metro counterparts (RR=1.06, 95%CI: 1.04-1.09) |
| Consedine et al.^[54]^  US | 2009 | Jamaican and Trinidadian Tobagonians had lower odds of undergoing annual PSA tests than US-born European Americans | Jamaican (aOR=0.42, 95%CI: 0.21–0.86) and Trinidadian Tobagonians (aOR=0.25, 95%CI: 0.12–0.53) had lower odds of annual PSA testing compared to US-born European-Americans. |
| Malika et al.^[70]^  US | 2021 | Caribbean immigrants received PSA testing more likely than African Americans | 58.4% of African Americans, 56.8% of Caribbean immigrants, and 42% of African immigrants underwent PSA testing in the past. At the same time, 49.3% of African Americans, 46.4% of Caribbean immigrants, and 36.7% of African immigrants underwent DRE in the past. |
| Loeb et al. ^[88]^  Sweden | 2013 | Immigrant men were significantly less likely to be diagnosed through PSA testing compared to Swedish-born men | Non-native men were less likely to be diagnosed with PCa through PSA testing (aOR=0.58, 95% CI: 0.55–0.62). There were differences by regions of birth; males from Asia, Africa, south and Central America, and Europe had lower odds of PCa screening (aOR=0.58, 95%CI: 0.55-0.62). |
| Lee et al. ^[46]^  US | 2011 | African American, Jamaican, and Trinidadian and Tobagonian were less likely to maintain annual PSA testing. | Compared to US-born whites, Jamaican (aOR=0.3, 95% CI: 0.15-0.69) and Trinidadian and Tobagonian (aOR=0.2, 95%CI: 0.08-0.39) men were less likely to undergo annual PSA screening |
| Kudadjie et al.^[66]^  US | 2008 | Compared to European Americans, Eastern Europeans, Haitians, Puerto Ricans, and African Americans, less frequent PSA tests and DRE in 10 years. | Compared to European Americans, Eastern Europeans, Haitians, Puerto Ricans, and African Americans, less frequent PSA screening and DRE in 10 years. The average number of DREs was 1.6, 2.7, and 2.3 for these groups, respectively, compared to 3.1 for European-American men (p<0.05). |
| Martins et al. ^[80]^  2021 | 2021 | Black men were less likely to be offered DRE than white men. | Significantly fewer black patients being offered DRE during their first consultation with a doctor compared to whites (72% vs 42%, p=0.02). In addition, Asians offered PCa screening less frequently than white men (36% vs 59%, aOR=0.39, 9%CI: 0.17-0.92; p=0.03). |
| Weber et al. ^[21]^  Australia | 2009 | Men from CALD backgrounds received PSA testing less often than Australian-born men | Compared to Australian-born, those from East Asia (aOR=0.41, 95%CI: 0.29–0.60) and those speaking non-English language (aOR=0.85, 95%CI: 0.75–0.96) had lower odds of undergoing a PSA test. |
| Lee et al.  ^[38]^  US | 2012 | African American and African-Caribbean men undergo annual DRE less frequently than white men | Compared to Whites (35.5%), African-Americans (16.9%; aOR=0.17, 95%CI: 0.07-0.37), Jamaican (26.5%; aOR=0.26, 95%CI: 0.12-0.57), Trinidadian and Tobagonians (19.5%; 0.16, 0.07-0.38) men had less often annual DRE than white men |
| Nair-Shalliker et al. ^[44]^  Australia | 2018 | Foreign-born men had lower odds of undergoing PSA testing compared to Australian-born men | Men from North America (aOR=0.73, 0.61-0.89), New Zealand (aOR=0.88, 95%CI: 0.79-0.99), and the UK (aOR=0.85, 95%CI: 0.80-0.89) had lower odds of PSA testing compared to Australian-born |
| Ilunga et al. ^[25]^  US | 2016 | Foreign-born men reported less frequent PSA tests compared to US-born men | Foreign-born Hispanics (29.1%), Asians (23.9%), Black/African-American (45.83%), and other ethnicities (25%) had lower PSA tests than US-born Whites (56.3%). Foreign-born Asians (aOR=0.22, 95%CI: 0.08-0.62) and blacks/African Americans (aOR=0.13, 95%CI: 0.05-0.33) associated with lower PSA test. |
| Dallo and Kindratt ^[55]^  US | 2015 | European-born non-Hispanic white men had lower odds of PSA testing than European-American men | 49% of European-born and 41% of Arab-born non-Hispanic whites were PSA-tested vs. 40% of US-born. After adjusting sociodemographic and acculturation factors, European-born non-Hispanic white men had lower odds (aOR=0.47, 95%CI: 0.85) of PSA testing than US-born men, but not Arab men did not. |
| Johnson et al.^[64]^  US | 2020 | Foreign-born men received PSA tests less often compared to US-born men | Foreign men were less likely to get PSA testing than US-born men (52.5% vs 35.8% in 2010 and 54.0% vs 33.2% in 2015). Foreign-born males were less likely than US-born men to have ever had a PSA test (aOR=0.82, 95%CI: 0.70-0.95, p=0.008). |
| Li et al. ^[67]^  US | 2021 | Affordable Care Act has been shown to improve the uptake of preventive health services among immigrants. | Immigrants who gained new insurance coverage following the Affordable Care Act were shown to improve PSA testing uptake by 11.78% compared to US-born insured men (p=0.0190) |
| Magin et al. ^[19]^ Australia | 2017 | Men from non-English speaking backgrounds had lower odds of receiving an asymptomatic PSA test | General practitioners order asymptomatic PSA testing less often for men from non-English speaking backgrounds compared to those from English speaking backgrounds (aOR=0.40, 95%CI: 0.19, 0.86, p=0.018) |
| Stone, Labban et al. ^[20]^  US | 2024 | Men with limited English proficiency were associated with lower odds of lifetime PSA testing | Men who reported not speaking English at all had significantly lower ever have had (lifetime) PSA screening compared to men who speak English very well (58.4% vs 79.6%; aOR= 0.56; 95% CI 0.35–0.91, p=0.019) |
| Wallner et al. ^[50]^  US | 2012 | Men who spoke a language other than English PSA tested less frequently than their English-speaking counterparts. | Men who spoke Spanish (22.71%) and Asian languages (25.2%) had lower PSA testing than English-speaking men (28.97%); p< 0.001 |
| McGuiness et al. ^[85]^  Australia | 2017 | Men who spoke languages other than English at home were less likely to undergo screening tests for PCa | Men who preferred to speak languages other than English at home had lower screening participation (DRE=38.5% vs 63.2%; PSA=56.3% vs 74.7%). |
| Cobran et al. ^[24]^  US | 2014 | No significant difference observed between Caribbean-born and US-born men | Caribbean-born blacks had a higher PCa fatalism score compared to US-born blacks (p=0.02). However, there was no statistically significant difference was found in PSA testing between US-born and Caribbean-born blacks (aOR=0.80, 95%CI: 0.26-2.48, p>0.05) |
| Odedina et al. ^[39]^  US | 2011 | There is no significant difference in the PSA test between foreign-born and US-born men | Chemoprevention and PCa-risk reduction discussions differed between subgroups. PSA testing (32.56%, US-born vs 31.45%, African-born, 30.1%, Caribbean-born, p = 0.668) and DRE (28.2%, US-born vs 29.4%, African-born vs 25%, Caribbean-born, p=0.422) were not statistically different between US-born, African-born, and Caribbean-born blacks. |
| Valdovinos et al. ^[76]^  US | 2016 | No significant difference in PSA test adherence and screening utilisations between foreign-born and US-born Hispanic/Latino. | Among Hispanic/Latino men aged 50–74, 30.1% were checked for PSA but did not adhere the guideline, whereas 28.9% were not PSA screened at all. There was no statistically significant difference in PSA screening that was not compliant to the guidelines (aOR=1.54, 95%CI: 0.62, 3.86) or PSA screening at all (aOR=1.64, 95%CI: 0.72, 3.71) based on nativity status |
| Hei et al.^[62]^  US | 2019 | Chinese Americans had less involvement in PSA testing, which was influenced by years lived in the US and the level of neighbourhood cohesion | 18.5-28.5% of Chinese Americans have ever had PSA tests with or without neighbourhood cohesion. Being born in China (aOR=0.59, 95%CI: 0.35–0.98) and living in medium neighbourhood cohesion is associated with increased use of the PSA test (aOR=1.60, 1.14-2.24), duration of stay (aOR=1.02, 95%CI: 1.00-1.03), preferred language (aOR=0.42, 95%CI: 0.29-0.62) |
| Ma et al. ^[69]^  US | 2012 | Asians had less access to PSA testing; only 21.8% of these men ever tested for PCa screening. | 78.2% of Asians (56.72% China, 88% Vietnamese, 96.97% Cambodian, and 78.85% Korean) had never undergone PSA testing screened PSA testing. Among those tested, only 14.3% (ranged 2.0% to 25%) underwent PSA testing in 12 months. |
| Glenn et al. ^[59]^  US | 2009 | South Asian men had a lower uptake of PSA testing for PCa screening, with only one in five undergoing PSA testing with guidelines. | South Asian men (Indian, Pakistani, Bangladeshi, and Sri Lankan) have a lower lifetime prevalence of PSA testing, ranging from 17% to 40%. Of these, 5-20% received PSA testing that adhered to guidelines. |
| Kleier ^[65]^  US | 2020 | Perceived susceptibility among Haitian-Americans associated with increased uptake of PCa screening | Haitian-Americans who had perceived susceptibility to PCa were more likely to undergo PCa screening and examination (p <0.05) |
| Lee and Jung ^[26]^  US | 2021 | 40% of Korean immigrants in the US aged 50 years or older had never screened for PSA in the past. | 59.79% of Korean immigrants reported a history of PSA testing, with 66% of men having undergone screening within 12 months. |
| Lepore et al.^[47]^  US | 2017 | More than half of black immigrants in the US underwent PSA screening without their awareness | Only 46.5% of black immigrant men were aware of their PSA testing. Men with lower levels of education had increased odds of undisclosed screening compared to those who attended college or post-secondary school (aOR=4.37, 95%CI: 1.723-10.970). |
| Lepore et al.^[48]^  US | 2017 | Tailored interventions for immigrants have been shown to improve PCa knowledge and minimise decisional conflict, but no effect on PSA testing. | Approximately one-fourth of men reported that they had had PSA testing (27.8%) or DRE (24.4%) in the past than 12 months or more. |
| Ma et al. ^[68]^  US | 2009 | Only 43% of Chinese Americans in the US had PSA testing for PCa screening | 56.7% of Chinese Americans were never screened for PSA testing, which was influenced by sociocultural factors. |
| Moreno et al.^[27]^  US | 2012 | Adherence to PCa screening is a concern and is influenced by interplaying factors, including the degree of acculturation. | Hispanics with a higher degree of U.S. American social relations (acculturation score) were positively associated with adherence to prostate cancer screening (aOR=1.52, 95% CI: 1.01–2.28, p=0.05) |
| Tim Wong  ^[86] a^  Australia | 2014 | CALD men in Australia had unmet PCa health information, and nearly more than half of the men were not getting PSA tested. | Nearly two-thirds of men from CALD backgrounds in Australia had unmet needs for health information language other than English. Only, 43% of men from CALD backgrounds had PSA testing in life time, of whom 29% of men PSA tested in past 12 months. |

Abbreviations: aOR: adjusted odds ratio, CALD: culturally and linguistically diverse backgrounds, CI: Confidence Interval, DRE: Digital rectal examination, PCa: Prostate cancer PSA: Prostate-specific Antigen, RR: Relative Risk, US: United States

Supplementary Table 3: Summary of statistical estimates and differences regarding prostate cancer treatment modalities (n=12 studies)

| Author (s), publication year, Country | | Key findings | Brief summary of key statistics |
| --- | --- | --- | --- |
| Jain et al.^[63]^  US | 2022 | Disparities in PCa treatment were observed received among Asian ethnicities. | Compared to Whites, Japanese were more likely to receive active treatment or active surveillance compared to Whites (aOR=1.46, 95%CI: 1.09-1.97, p value=0.013) |
| Kum et al.^[81]^  UK | 2021 | African/Afro-Caribbean men on AS for low-risk PCa were more likely to be non-adherent to scheduled appointments compared to Caucasians or others. | 25% of African/Afro-Caribbean men had missed follow-up appointments [≥20%] compared to only 10% of Caucasians or others. African/Afro-Caribbean ethnicity is associated with higher odds of non-adherence to AS follow-up (aOR=2.77, 95%CI: 1.56-4.91, p<0.0001). |
| Estrada-Mendizabal et al. ^[57]^  US | 2024 | Despite being more likely to present with advanced disease at diagnosis, Mexican men had lower odds of receiving treatment compared to non-Hispanic White men. | Hispanics underwent External Beam Radiation Therapy (EBRT) more frequently than Non-Hispanic Whites (NHW); 46% of Cubans and 39% of Puerto Ricans versus 26% of NHW received EBRT. NHW patients received robot-assisted prostatectomy (RAP) more often than Hispanics: 37% in NHW versus 23% in Cubans and 26% in Puerto Overall, Mexicans had lower odds of receiving treatment compared NHW (aOR=0.68, 95%CI: 0.54-0.85, p< 01) |
| Fedewa et al .^[58]^  US | 2013 | Differences in radical prostatectomy treatment utilisation were observed between US-born vs Jamaican- born and West-African-born men. | A greater percentage of West-African-born men (39.02%) received radical prostatectomy compared to US-born men (23.76%-32.39%; p <0.01) |
| Swami N et al.^[75]^  US | 2023 | Treatment for unfavourable intermediate-risk and high-risk PCa varies significantly with country of birth and race/ethnicity. | Despite their increased risk of presentation with high-risk disease, Hispanics of Mexican origin (0.62, 95%CI: 0.50–0.79) and non-Other specified (NOS) (aOR=0.55, 95%CI: 0.50–0.61) had lower access to PCa treatment. |
| Heard et al.^[61]^  US | 2022 | Foreign-born men with intermediate PCa were more likely to choose surgery compared to US-born men. | 40% of foreign-born and 23% of US-born men received surgery, whereas 50% of US-born men and 35% of foreign-born men received radiation therapy. Being foreign-born was associated with more likelihood of choosing surgery compared to being US-born (aOR= 18.7, 95%CI: 1.58–220.9; p=0.020) |
| Xu et al. ^[78]^  US | 2015 | The proportion of radical prostatectomy was slightly higher among foreign-born Asians than US-born Asians. | Overall, radical prostatectomy was slightly higher among foreign-born Asians compared to their US-born counterparts. On stratified analysis, foreign-born Japanese had the highest proportion of radical prostatectomy (37%), whereas foreign-born Filipinos had the lowest proportions (30%). |
| Smigelski et al.^[74]^  US | 2021 | Patients from the boroughs (neighbourhoods) with a higher percentage of foreign-born residents were more likely to receive aggressive local therapies. | Men from neighbourhoods with a higher proportion of foreign-born residents were more likely to receive aggressive local therapies (aOR=4.84, 95%CI: 2.36-9.90). |
| Patel et al.^[73]^  US | 2013 | South Asian men had worse pathological profiles than the US general population undergoing radical prostatectomy. | South Asian men had a worse pathological profile than the US general population undergoing radical prostatectomy (positive surgical margin 26% vs 24%) |
| Thogersen et al.^[87]^  Norway | 2020 | Threre was no statistically significant difference in PCa treatment received between Norwegian-born and Western and non-Western immigrants. | Among men aged <75 years with locoregional PCa, non-western immigrants had no statistically significant difference in the use of surgery (aOR=0.75, 95%CI: 0.49-1.16), radiation therapy (aOR=0.89, 95%CI: 0.73-1.08), and RAP (aOR=1.14, 95%CI: 0.90-1.43) compared to Norwegian men. Similarly, western immigrants exhibited no significant differences in treatment pattern: surgery (aOR=0.91, 95%CI: 0.79-1.06), radiation therapy (aOR=1.05, 95%CI: 0.87-1.26), and RAP (aOR=1.17, 95%CI: 0.85-1.61). |
| Chu et al. ^[51]^  US | 2015 | No statistically significant differences were observed in choosing AS and immediate treatment for treatment for low-risk PCa. based on patients’ preferred language | 13.5% of the immediate treatment and 9.7% in the active surveillance group were patients of non-English speakers. No statistically significant difference was reported in choice of AS based on language (OR=1.31, 95%CI: 0.65-2.61; p=0.447). |
| Eren M.F et al. ^[90]^  Turkey | 2023 | Most Syrian refugees presented with advanced-stage disease, received suboptimal treatment, and had higher rates of treatment non-compliance | Nearly two-thirds (64%) of refugee patients with PCa in Turkey presented with advanced disease at diagnosis. Most of the patients received suboptimal treatment, with only 20% of patients receiving androgen deprivation therapy. Treatment non-compliance rate was high, with 42% of the refugees were non-compliant to the prescribed radiation therapy sessions. |

aOR: Adjusted Odds Ratio, AS: Active surveillance, EBRT: External Beam Radiation Therapy, NHW: Non-Hispanic Whites, PC: Prostate cancer, US: United States
